# PINK1 kinase dysfunction triggers neurodegeneration in the primate brain without impacting mitochondrial homeostasis

**DOI:** 10.1007/s13238-021-00888-x

**Published:** 2021-11-20

**Authors:** Weili Yang, Xiangyu Guo, Zhuchi Tu, Xiusheng Chen, Rui Han, Yanting Liu, Sen Yan, Qi Wang, Zhifu Wang, Xianxian Zhao, Yunpeng Zhang, Xin Xiong, Huiming Yang, Peng Yin, Huida Wan, Xingxing Chen, Jifeng Guo, Xiao-Xin Yan, Lujian Liao, Shihua Li, Xiao-Jiang Li

**Affiliations:** 1 Guangdong Key Laboratory of Non-human Primate Research, Guangdong-Hongkong-Macau Institute of CNS Regeneration, Jinan University, Guangzhou, 510632 China; 2 Key Laboratory of Brain Functional Genomics of Ministry of Education, Shanghai Key Laboratory of Brain Functional Genomics, and Shanghai Key Laboratory of Regulatory Biology, School of Life Sciences, East China Normal University, Shanghai, 100021 China; 3 Department of Neurology, Xiangya Hospital, Central South University, Changsha, 410008 China; 4 Department of Anatomy and Neurobiology, Xiangya School of Medicine, Central South University, Changsha, 410008 China

**Keywords:** Parkinson’s disease, neurogenesis, neurodegeneration, mitochondria, non-human primates

## Abstract

*In vitro* studies have established the prevalent theory that the mitochondrial kinase PINK1 protects neurodegeneration by removing damaged mitochondria in Parkinson’s disease (PD). However, difficulty in detecting endogenous PINK1 protein in rodent brains and cell lines has prevented the rigorous investigation of the *in vivo* role of PINK1. Here we report that PINK1 kinase form is selectively expressed in the human and monkey brains. CRISPR/Cas9-mediated deficiency of PINK1 causes similar neurodegeneration in the brains of fetal and adult monkeys as well as cultured monkey neurons without affecting mitochondrial protein expression and morphology. Importantly, *PINK1* mutations in the primate brain and human cells reduce protein phosphorylation that is important for neuronal function and survival. Our findings suggest that PINK1 kinase activity rather than its mitochondrial function is essential for the neuronal survival in the primate brains and that its kinase dysfunction could be involved in the pathogenesis of PD.

**Supplementary Information:**

The online version contains supplementary material available at 10.1007/s13238-021-00888-x.

## INTRODUCTION

PTEN induced putative kinase 1 (PINK1) was found to be a mitochondrial serine/threonine protein kinase that can phosphorylate and recruit Parkin, a cytosolic E3 ubiquitin ligase, to damaged mitochondria and to mediate the ubiquitination of surface proteins of mitochondria, resulting in removing defective mitochondria by autophagy. This process is known as mitophagy in protection of neuronal degeneration and maintenance of healthy mitochondria following cellular injury and stress (de Vries and Przedborski, 2013; Pickrell and Youle, 2015; Chu, 2019). In support of the involvement of PINK1 and Parkin in mitophagy, mutations in the human *PINK1* and *Parkin* gene were found to result in autosomal recessive Parkinson’s disease (PD) that is featured by neurodegeneration in association with mitochondria dysfunction (Valente et al., 2004; McInerney-Leo et al., 2005; Corti et al., 2011; Pickrell and Youle, 2015). Based on the primary role of PINK1 in mitophagy and the genetic mutations of *PINK1* that may cause loss of function, a number of *PINK1* knockout animal models have been established for investigating PD pathogenesis. However, these *PINK1* KO animal models have not validated the important *in vitro* findings for the function of PINK1 in mitophagy (Whitworth and Pallanck, 2017; Cummins and Gotz, 2018) or recapitulated selective and overt neurodegeneration seen in PD (Kitada et al., 2007; Gispert et al., 2009; Xiong et al., 2009; Akundi et al., 2011; Zhou et al., 2015; Wang et al., 2016). In contrast, recent studies using Mito-QC mouse and Drosophila models demonstrated that basal mitophagy activity is not affected by the loss of PINK1 (Lee et al., 2018; McWilliams et al., 2018). Thus, the primary deficit in the mammalian brains caused by *PINK1* mutations remains elusive.

The biggest obstacle to address the *in vivo* role of PINK1 in the brain stems from the difficulty in detecting endogenous PINK1 at the protein level in rodent brains and cell lines. Endogenous PINK1 in the mouse brain is expressed at a very low level and can only be detected via immunoprecipitation (McWilliams et al., 2018). *In vitro* studies revealed that PINK1, once imported into the inner membrane of mitochondria, is rapidly cleaved by proteases within the mitochondria, followed by proteasomal degradation (Yamano and Youle, 2013). Thus, most studies of endogenous PINK1 rely on *in vitro* depolarizing mitochondria that can stabilize PINK1 on the mitochondria (Narendra et al., 2010) and led to the theory that the major function of PINK1 is to maintain mitochondrial quality and clear damaged mitochondria. However, this theory remains to be validated using an animal model in which loss of PINK1 can faithfully replicate selective neurodegeneration in PD.

Our recent studies demonstrated that genetically modified large animal models could more closely mimic neuropathology seen in patient brains. For example, Huntington disease (HD) knock-in pigs show striking and selective neurodegeneration in the pig brain, which is not seen in HD knock-in mice (Yan et al., 2018). However, previous studies have generated *PINK1* knockout pigs but did not report any behavioral phenotypes and neurodegeneration in these pigs (Zhou et al., 2015; Wang et al., 2016). Using non-human primates to target the *PINK1* gene via CRISPR/Cas9, our recent studies revealed that depletion of PINK1 in monkey embryos causes severe neurodegeneration in the monkey brain (Yang et al., 2019a, b). These findings raised important questions as to why and how PINK1 loss can selectively cause neurodegeneration in the primate brain.

In the current study, we found that the PINK1 kinase, rather than its full-length form that is associated with mitochondria, is selectively expressed in the brain tissues of humans and monkeys. Importantly, loss of PINK1 in cultured monkey neurons and in the monkey brains at different ages causes neuronal degeneration, indicating an essential role of PINK1 kinase in neuronal survival in the primate brain. However, deficiency in PINK1 did not alter mitochondrial morphology and dynamics. Instead, loss of PINK1 in the monkey brain caused a significant reduction of phosphorylation of a number of proteins important for neuronal function and survival. Further, PINK1 kinase phosphorylation deficiency also occurs in human cells harboring PINK1 mutations. Our findings demonstrate for the first time that PINK1 kinase activity rather than its mitochondrial function is selectively essential for neuronal survival in the primate brain and suggest that PINK1 kinase dysfunction may be involved in PD and other pathological conditions in humans.

## RESULTS

### Selective expression of PINK1 kinase in the human and monkey brains

Our recent study showed a remarkable reduction of PINK1 protein in CRISPR/Cas9-targeted monkeys (Yang et al., 2019b), which is apparently different from the previous findings in PINK1 knockout rodent models, as none of these rodent models were able to demonstrate the difference in PINK1 protein expression between wild type and *Pink1* knockout animals (Kitada et al., 2007; Gispert et al., 2009; Xiong et al., 2009; Akundi et al., 2011). Whole genome sequencing verified the specific targeting of the *PINK1* gene in mutant monkey brains (Yang et al., 2019b). In order to compare the expression levels of endogenous PINK1 in mouse and monkey brains, we also generated a new *Pink1* KO mouse model by disrupting exon 2 and exon 4 in mouse *Pink1* via CRISPR/Cas9 (Fig. S1A and S1B) with the same strategy for deleting the monkey *PINK1* (Yang et al., 2019b). We obtained multiple mouse founders with different types of PINK1 mutations (Fig. S1C). Of these mutant mice, mice carrying the targeted exon 2 (Δ38/Δ38) and exon 4 (Δ7/Δ7) are homozygous *Pink1* KO mice (Δ38/Δ38; Δ7/Δ7), which were found to have disrupted two alleles of the *Pink1* gene (Fig. S1C) and used for further characterization. Although the new *Pink1* KO mice lived normally without any obvious phenotypes or neurodegeneration, similar to other previously reported *Pink1* KO mice, they provided a rigorous control to validate the expression of Pink1 in mice.

**Figure 1:**
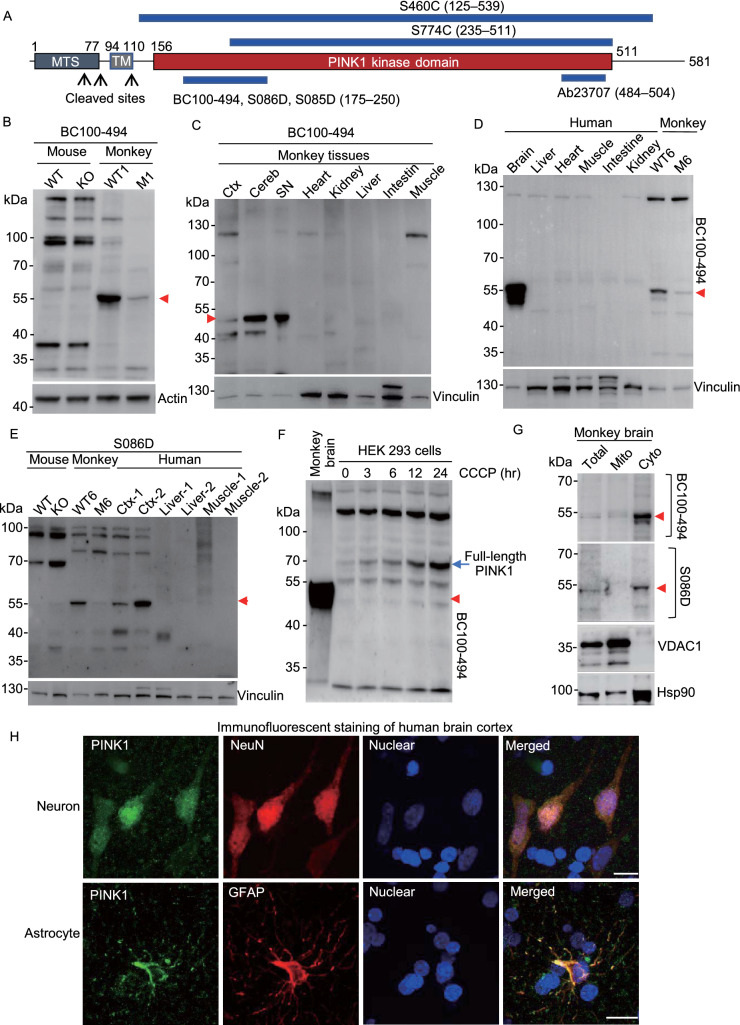
**Selective expression of PINK1 in the primate brain**. (A) Six PINK1 antibodies used and their epitopes. MTS: a mitochondrial-targeting sequence; TM: transmembrane domain. (B) BC100-494 Western blot analysis of brain cortical (Ctx) lysates of wild-type and *Pink1* homozygous KO mouse, *PINK1* mutant (M1) monkey, and wild-type monkey (WT1). Note the absence of specific Pink1 expression in the mouse brain. (C) BC100-494 Western blot of monkey brain regional (cortex: Ctx; cerebellum: Cereb; substantia nigra: SN) and peripheral tissues. (D) BC100-494 Western blot analysis of the brain stem and peripheral tissues from the 54 year old individual. WT and M6 monkey cortical tissues were included to indicate that PINK1-55, which is reduced in M6, is selectively expressed in the human brain. (E) S086D Western blot of WT, *Pink1* KO mouse brain cortex, brain cortical tissues of wild type (WT6) and mutant (M6) monkeys, brain cortical tissues (Ctx-1 and Ctx-2) and tissues of muscle and liver from two human individuals. (F) BC100-494 is able to recognize the full-length PINK1 in human HEK293 cells treated with 10 μmol/L CCCP for 3–24 h, and PINK1-55 is smaller than full-length PINK1. (G) In the monkey brain, PINK1-55 is enriched in the cytoplasm (Cyto). VDAC1 is a mitochondrial (Mito) protein, and Hsp90 is a cytosolic protein. (H) Double immunostaining of the postmortem brain from a 51-year-old individual showing PINK1 is present in NeuN- and GFAP-positive cells. Scale bars: 10 μm

Using RT-PCR with primers specific for exons 1–2 and 6–7 (Fig. S1D), we found that *PINK1* transcripts were undetectable in the brain cortical tissues of *Pink1* KO mice (Fig. S1E and S1F), suggesting a complete knock-out of the mouse *Pink1*. In a *PINK1* targeting monkey (M1), which was generated in our recent study (Yang et al., 2019a, b), different degrees of reduction of transcripts of exon 1–2 and exon 6–7 were seen (Fig. S1E and S1F), consistent with the mosaic PINK1 targeting by CRISPR/Cas9. We then used six commercially available antibodies (rabbit BC100-494, sheep S086D, sheep S085D, rabbit Ab23707, sheep S460C, and sheep S774C) that recognize different regions in PINK1 (Figs. 1A and S2A) to examine PINK1 expression in the mouse, monkey, and human tissues. Because all PINK1 antibodies label some non-specific bands, it is necessary to use the mutant monkey brains (M1 and M6) that were targeted on the *PINK1* gene (Yang et al., 2019b) to verify the specificity of the PINK1 antibodies. CRISPR/Cas9 targeting created mosaic mutations that do not completely delete the *PINK1* gene but could reduce its expression. The rabbit antibody BC100-494, which is against human PINK1 (175–250 aa), appeared to react strongly with PINK1 at ~55 kDa (PINK1-55) that was markedly reduced in the M1 monkey brain, indicating that PINK1-55 is a protein product of the targeted *PINK1* gene (Fig. 1B). However, BC100-494 did not detect any specific band that was only present in the wild type mouse brain and absent in *Pink1* KO mouse brain (Fig. 1B), supporting the idea that endogenous Pink1 in the mouse brain is expressed at an undetectable or very low level. Comparison of the monkey brain and peripheral tissues showed that PINK1-55 was selectively expressed in the brain tissues (Fig. 1C). This brain selective expression was validated by comparing wild type and mutant monkey (M6) tissues, showing that PINK1-55 was specifically expressed in the monkey brain (Fig. S2B). The selective expression of PINK1 in the primate brain was further verified by Western blot of human peripheral and brain tissues and additional *PINK1* mutant monkey (M6) that showed a reduction in PINK1-55 expression because of *PINK1* targeting (Fig. 1D). We also used a sheep antibody (S086D) against mouse Pink1 (175–250 aa) and obtained similar results that additionally revealed the selective brain expression of PINK1-55 in human tissues (Fig. 1E). To verify that PINK1-55 is only present in the primate brain, we also used three other antibodies (S774C, Ab23707 and S460C) for Western blot. The results demonstrated that all these antibodies could detect PINK1-55 in the human and monkey cortex and that PINK1-55 was reduced in the M6 monkey brain (Fig. S2C). Again, none of these antibodies could detect a specific band that was only present in wild type mice (Fig. S2C). We further used five anti-PINK1 antibodies to compare PINK1-55 expression in different monkey brain regions. The results consistently demonstrated that PINK1-55 was expressed at a higher level in the substantia nigra (SN) (Fig. S2D). Given that the SN is the most vulnerable brain region in PD, the unique expression pattern of PINK1 in the primate brain regions is consistent with its involvement in the selective neurodegeneration in PD.

Comparing different antibodies revealed that BC100-494 reacted strongly with PINK1-55 in the primate brain so that we used BC100-494 for further characterization of PINK1 expression. We found that PINK1-55 is undetectable or at the very low level in several cell lines (Fig. S2E). BC100-494 has been used to detect full-length PINK1 at ~70 kDa, which is localized to damaged mitochondria in human and mouse cell lines (Okatsu et al., 2012). Indeed, BC100-494 was able to recognize full-length PINK1 at ~70 kDa, which was induced by a mitochondrial membrane potential disruptor, CCCP (carbonyl cyanide 3-chlorophenylhydrazone), in human (HEK293) and mouse (N2A) cell lines and is larger than PINK1-55 (Figs. 1F and S2F). These results indicate that PINK1-55 lacks the N-terminal PINK1 region that can associate with the mitochondria. Consistently, fractionation of wild-type monkey brain tissues revealed that PINK1-55, which was detected by both BC100-494 and S086D, is mainly present in the cytoplasm (Fig. 1G).

Using isolated white matter that is enriched in glial cells and gray matter that is enriched in neurons for Western blot, we found that PINK1-55 is expressed in both neuronal and glial cells (Fig. S3A). Immunocytochemical staining revealed abundant distribution of PINK1-55 in the cytoplasm of neuronal and glial cells (Fig. S3B and S3C). Using the postmortem brain tissues from humans, we also found that PINK1 was detected in neurons and astrocytes (Fig. 1H). Taken together, PINK1-55 is a cytoplasmic form of PINK1 that is selectively expressed in the human and monkey brains.

### Acute deletion of PINK1 in the adult monkey brains causes neurodegeneration

**Figure 2:**
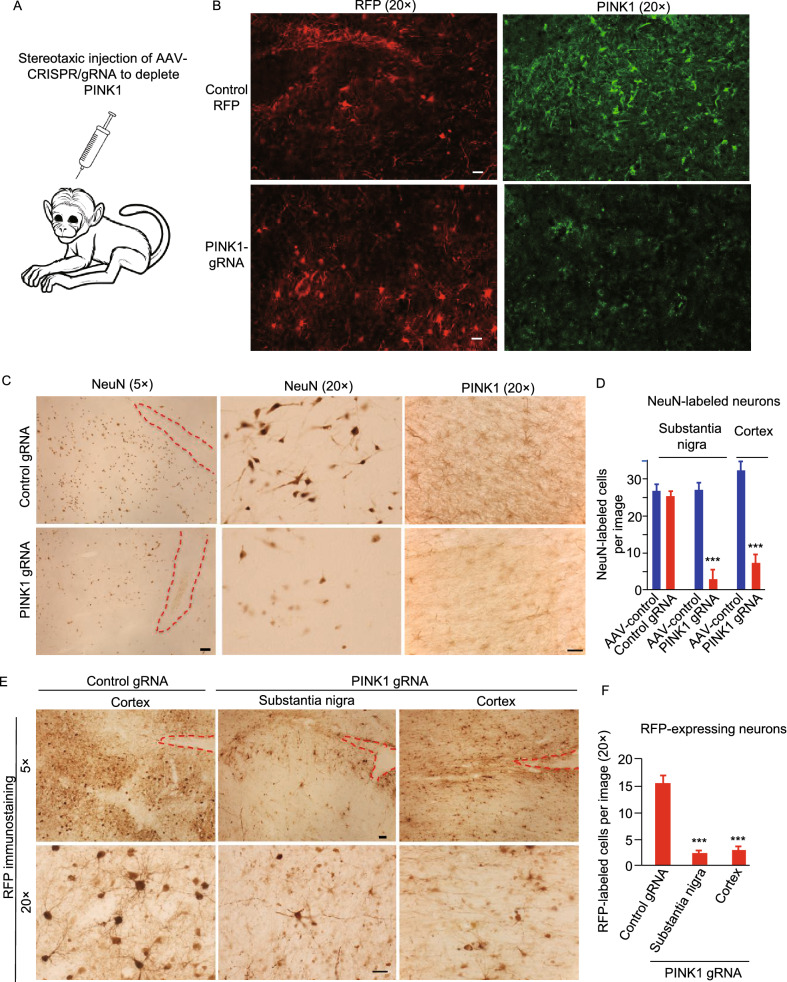
**Loss of PINK1 causes neurodegeneration in the adult monkey brain**. (A) Stereotaxic injection of AAV-PINK1 gRNA-RFP/Cas9 into the monkey brain region to target the monkey *PINK1* gene. Control gRNA-RFP with AAV-Cas9 served as a control. AAV9 serotype was used. (B) Double immunofluorescent staining confirmed the reduction of PINK1 (green) in the monkey cortex by AAV-PINK1 gRNA-RFP/Cas9 injection. Scale bar: 20 μm. (C) AAV viral expression of CRISPR/Cas9 to target the *PINK1* gene in the substantia nigra and prefrontal cortex in wild type adult monkeys (WT11, 3-year-old, WT12, WT13, 12-year-old, and WT14, 10-year-old) resulted in remarkable degeneration of neuronal cells, which is evident by the reduced number of NeuN-positive cells. Red lines indicate the injection sites that are visible as needle tracks. Scale bars: 20 μm. (D) Quantitative assessment of NeuN-positive neurons in the AAV-control (AAV-GFP), AAV-Cas9/Control-gRNA, or AAV-Cas9/PINK1-gRNA injected substantia nigra and prefrontal cortex. (E) Representative immunostaining micrographs show a remarkable decrease in RFP-expressing neurons that express either AAV-Cas9/Control-gRNA/RFP or AAV-Cas9/PINK1-gRNA/RFP. Scale bars: 20 μm. (F) Quantitative assessment of RFP-expressing neurons in (E). In (D) and (F), the data are presented as mean ± SEM and were obtained by counting cells in 17–21 fields (20×) from each injection group (*n* = 3 animals per group). ***P < 0.001

Our recent studies have shown that targeting the *PINK1* gene in embryos leads to neuronal loss in monkey brains (Yang et al., 2019a, b), therefore, next we wanted to know whether directly removing PINK1 in the brains of wild-type adult monkeys could cause neurodegeneration. We previously used stereotaxic injection of AAV9 vector expressing CRISPR/Cas9 and gRNA (AAV-gRNA) to target the mutant huntingtin (*HTT*) gene in adult mouse brain, which efficiently and permanently eliminated mutant HTT-mediated neuronal toxicity without affecting neuronal viability (Yang et al., 2017). We used the same AAV-gRNA vector to target *PINK1* exon 2 and exon 4 in the monkey brain (Figs. 2A, S4A and S4B) and performed stereotaxic injection of this viral vector into the brains of rhesus monkeys using the same method in our previous studies (Yang et al., 2015). Because AAV-gRNA also expressed RFP, immunohistochemistry with anti-RFP was able to detect RFP signal in the injected area one month after injection (Fig. S4C). Using Western blot with BC100-494, we confirmed the reduction of PINK1-55 in the AAV-PINK1 gRNA/Cas9-injected monkey cortex, and this reduction was also revealed by a different sheep anti-PINK1 antibody (S085D) (Fig. S4D).

To examine whether PINK1 deletion in adult monkey brains also causes neurodegeneration in different brain regions, we injected AAV-PINK1 gRNA/Cas9 into the prefrontal cortex and substantia nigra of adult monkeys (one 3-year-old, two 12-year-olds, and one 10-year-old) and then isolated their brain tissues 3 months later for histological examination. We first verified that targeting PINK1 could reduce PINK1 expression by performing double immunofluorescent staining of the injected monkey brain regions with antibody to RFP to detect *PINK1* gRNA expression and BC100-494 to detect PINK1. Targeting *PINK1* by AAV PINK1-gRNA/Cas9 apparently significantly reduced PINK1 expression as compared with the AAV control-gRNA/Cas9 injection (Fig. 2B). High magnification micrographs showed that AAV-infected neuronal cells, which showed both RFP and NeuN expression, were present in the AAV control-gRNA/Cas9-injected brain cortex but were rarely seen in the AAV-PINK1-gRNA/Cas9-injected cortex (Fig. S5A and S5B). We then used antibodies to NeuN, PINK1 and RFP to further assess neuronal loss in the injected monkey brains (Fig. 2C–F). We found that *PINK1* depletion in the cortex and substantia nigra in adult monkeys resulted in a remarkable loss of neuronal cells, evidenced by decreases in both NeuN staining (Fig. 2C and 2D) and RFP labeling (Fig. 2E and 2F) in the AAV-PINK1-gRNA/Cas9-injected area. However, no evident degeneration was found in AAV-GFP or AAV-control-gRNA/Cas9-injected brain regions (Fig. 2C and 2E). The selective neuronal loss in the AAV-PINK1-gRNA/Cas9-injected area was also supported by the abnormal and difficult movement of the left limbs of the injected monkey (Video S1), which is controlled by the contralateral right side of the substantia nigra that was injected with the AAV-PINK1-gRNA/Cas9, when compared with the right limbs that are controlled by the left substantia nigra injected with AAV-control gRNA/Cas9.

### Loss of PINK1 did not alter mitochondrial proteins and morphology in the monkey brain cells

**Figure 3:**
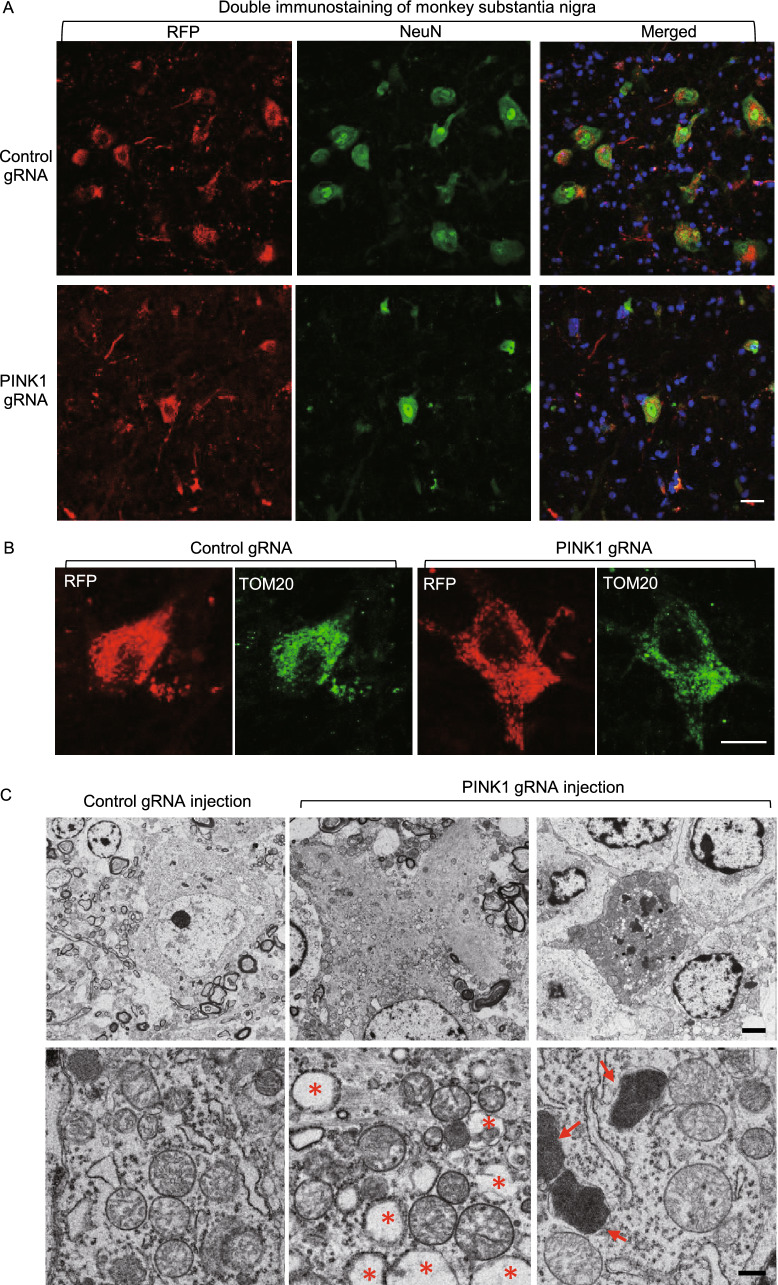
**Targeting PINK1 in the adult monkey brain caused neuronal loss without alteration in mitochondria**. (A) Targeting *PINK1* reduced NeuN expression in the AAV-PINK1 gRNA/Cas9-injected substantia nigra in the monkey brain. (B) In the *PINK1* targeted substantia nigra, the survived neurons showed that mitochondrial labeling by TOM20 antibody is not different from the control neuron without *PINK1* targeting. Scale bars: 10 μm. (C) Electron microscopy revealed degenerated cells in the substantia nigra injected with AAV-PINK1-gRNA/Cas9. Degenerated neurons show electron-dense cytoplasm, with no clear profiling or identifiable nuclear membrane (upper panel). Arrows indicate lysosomes, and stars indicate phagocytic vacuole-like structures. Mitochondrial morphology in degenerated neurons is not different from normal neurons in the control gRNA/Cas9-injected brain region (lower panel). Scale bars: 2 μm (upper panel), 0.5 μm (lower panel)

In the substantia nigra, we also found a remarkable reduction of large-sized and NeuN-positive neurons when PINK1 was depleted (Fig. 3A). Interestingly, in the survived neurons, immunostaining of the mitochondrial protein (TOM20) did not reveal any obvious alteration in the mitochondria density when comparing with control neurons infected by AAV-control gRNA/Cas9 (Fig. 3B). These results led us to use electron microscopy to explore whether loss of PINK1 can affect mitochondria. Degenerated neurons were seen in the substantia nigra that had been injected with AAV PINK1-gRNA/Cas9 when comparing with the AAV-GFP control (upper panel in Fig. 3C). The degenerated neurons displayed increased cytoplasmic density to dark profiling without clear nuclear and organellar structures and often contained increased lysosomal and phagocytic vacuole-like structures. Interestingly, in the degenerated neurons, the morphology and number of mitochondria appeared normal compared with the control gRNA-injected area (low panel in Fig. 3C). Quantification of ultrastructurally identifiable mitochondria also showed that the percentage (346 out of 387 or 89.4%) of mitochondria with normal morphology in AAV PINK1-gRNA/Cas9-injected substantia nigra was similar to that (279 out of 307 or 91.2%) in AAV-control gRNA/Cas9-injected substantia nigra.

**Figure 4:**
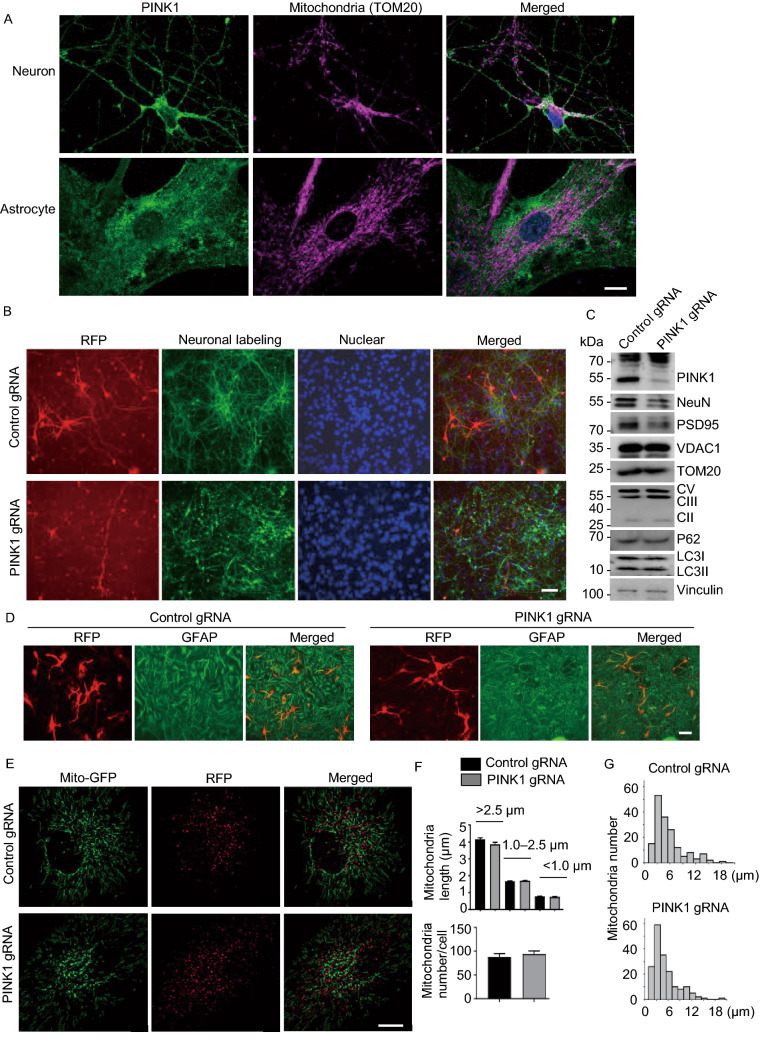
**Mitochondrial morphology and dynamics in PINK1 targeted cells**. (A) Double staining of cultured monkey neurons and astrocytes from a fetal monkey showing that PINK1 is not closely colocalized with the mitochondria protein TOM20. (B) Primary cultures from the fetal monkey were transfected by control gRNA-RFP or PINK1-gRNA-RFP with Cas9. Note a marked reduction of neuronal cells labeled by anti-Tuj1 after PINK1-gRNA-RFP transfection for 14 days. Scale bars: 20 μm. (C) Western blot confirmed that *PINK1* targeting of cultured monkey neuronal cells reduced the expression of PINK1 and neuronal proteins NeuN and PSD95, but not mitochondrial proteins (VDAC1, TOM20, Complex (C) II, V, and III), P62, LC3I, or LC3II. Vinculin served as a loading control. (D) *PINK1*-targeting does not affect the viability of cultured astrocytes that were labeled by anti-GFAP. Scale bar: 20 μm. (E) Mitochondrial dynamics in the primary cultured monkey astrocytes was assessed 14 d after transfection with control-gRNA RFP/Cas9 or PINK1 gRNA-RFP/Cas9. Cells were labeled with MitoTracker probes (Mito-GFP) and imaged by acquisition of multiple optical sections with an Olympus Spin SR confocal system. Scale bars: 5 μm. (F) Quantification of mitochondrial number and length. More than 1000 mitochondria per group live imaging acquisition were counted and manually measured for their lengths using OLYMPUS cellSens Dimension software. The data are presented as mean ± SEM. (G) The histograms showing the total track length distributions of mitochondria within 90 s in the control (top panel) and *PINK1* targeted glia cells (bottom panel)

Cultured monkey brain cells would allow us to monitor mitochondria dynamics. Thus, primary cultures were obtained from a fetal money brain cortex at embryonic day 90. Double immunostaining of the cultured monkey neuronal and glial cells at DIV14 did not show that PINK1 and the mitochondrial protein TOM20 were closely colocalized (Fig. 4A). The results are consistent with fractionation data (Fig. 1G) and suggest that the majority of endogenous PINK1 is at least not localized in mitochondria under physiological conditions. We then used electroporation to transfect primary cultures with PINK1-gRNA-RFP/Cas9 plasmids. After transfection for 14 days, we observed that many PINK1-gRNA-RFP/Cas9 transfected neurons (59.91% ± 12.11%, *n* = 145 cells) showed fragmented and short neurites, a degeneration phenomenon. In contrast, a small fraction of control transfected cells expressing control-gRNA-RFP/Cas9 (14.75 % ± 4.09%, *n* = 137 cells, *P* < 0.001 vs. PINK1 gRNA) were degenerated, perhaps because of electroporation, while the majority of control neurons developed long and intact neurites (Fig. 4B). Importantly, Western blot confirmed that deletion of the *PINK1* gene by its gRNA and Cas9 reduced the expression of PINK1 and neuronal proteins NeuN and PSD95. However, no obvious alterations in mitochondrial proteins (VDAC1, TOM20, Complex-II, -III, -V) were seen when compared with the control transfected neurons (Fig. 4C).

Because neuronal degeneration and cell death can affect mitochondrial dynamics and because PINK1 deficiency did not affect the survival of astrocytes, we used astrocytes to examine the impact of PINK1 deficiency on mitochondria dynamics. Consistent with the *in vivo* finding that PINK1 targeting did not obviously affect glial cells in the monkey brain (Fig. 2C–F), GFAP staining showed the normal morphology and numbers of astrocytes after *PINK1* targeting as compared with control astrocytes (Fig. 4D). We then used *PINK1*-targeted live astrocytes to measure mitochondrial dynamic changes in their size, lengths, and motility (Videos S2 and S3). This assay showed that mitochondrial dynamics in control and *PINK1*-targed astrocytes are similar (Fig. 4E–G).

**Figure 5:**
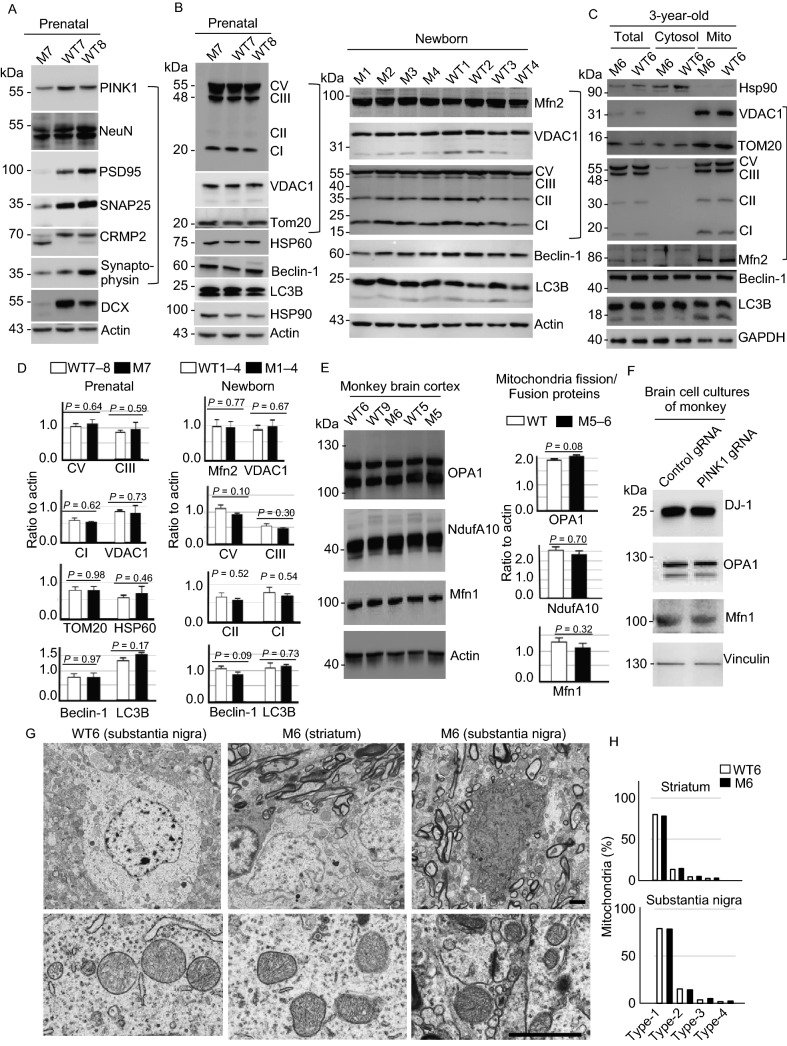
**PINK1 deficiency in fetal and young monkeys causes neurodegeneration without affecting mitochondrial proteins and morphology**. (A) Western blot analysis of the brain cortical tissues from a *PINK1* mutant fetal monkey (M7) and two fetal wild type (WT7 and WT8) monkeys. M7 monkey brain showed reduction in the expression of a number of neuronal (NeuN, PSD95, SNAP25, synaptophysin) proteins (Neu). (B) Western blot analysis of the fetal brain cortical tissues from M7 and WT7, WT8, newborn *PINK1* mutant (M1–M4) and wild type (WT1–WT4) monkeys. The results showed no any significant changes in the levels of mitochondrial proteins (Mito) including complex proteins (CI, CII, CIII, and CV) in mutant monkey cortex tissues when compared with age-matched control monkey tissues. The same protein samples were probed with various antibodies as indicated. (C) Western blot analysis of the cytosolic and mitochondrial fractions of brain cortical tissues from 3-year-old *PINK1* mutant (M6) and wild type (WT6) monkeys. No obvious alterations in the mitochondrial proteins (Mito) are seen in M6 monkey as compared with WT6. (D) Quantification of the relative levels of mitochondria related proteins (TOM20, VDAC1, CV, CI, CII, CIII) on the Western blots in (B). Ratios (mean ± SEM) of mitochondria related proteins to the loading control are presented. (E) The expression of mitochondrial proteins (OPA1, NduA10, and Mfn1) for fission/fusion is not affected by *PINK1* mutations in the monkey brain. (F) The expression of mitochondrial proteins for fission and fusion in primary cultures from a fetal monkey brain is not affected by *PINK1* mutations. (G) EM examination of the monkey brains of M6 and WT6. Low magnification micrographs (upper panel) and high magnification micrographs (lower panel) are presented. Scale bars: 2 μm. (H) The percentage of different types of mitochondria (Type I: normal appearing cristae; Type II: swollen, irregular or whirling cristae; Type III: discontinuous outer membrane or deficient cristae; Type IV: both discontinuous outer membrane and swollen cristae). Each group consists of 354–583 mitochondria examined

Our continuous study in generation of *PINK1* mutant monkeys yielded a prenatal *PINK1* targeted monkey (M7) that was aborted at the gestation days 135. Western blot analysis of the M7 brain tissues confirmed the reduction of PINK1 and neuronal proteins (Fig. 5A). However, no alterations in mitochondrial proteins were seen in the prenatal M7 monkey brain and newborn *PINK1* mutant monkey (M1–M4) brain tissues (Fig. 5B). Our early study also obtained a 3-year-old *PINK1* mutant monkey (M6) (Yang et al., 2019b), which provided us with enough fresh brain tissues for isolation of mitochondria fraction. Western blot analysis of the mitochondrial fraction isolated from the M6 monkey brain cortex showed that mitochondrial proteins were expressed at the similar levels as an age-matched WT monkey (WT6) (Fig. 5C). Quantitation of the ratios of mitochondria related proteins (TOM20, VDAC1, CV, CI, CII, CIII) to the loading control verified no difference between *PINK1* mutant and the age-matched control monkeys (Fig. 5D). We also performed Western blot to analyze the levels of proteins that participate in mitochondria fission/fusion (OPA1, NdufA10, and Mfn1) and found their levels were similar in wild type and *PINK1* mutant monkey cortex (Fig. 5E). The primary cultures from the fetal monkey brain also showed no obvious changes in DJ-1, OPA1, and Mfn1 after PINK1 was knocked down by *PINK1* gRNA/Cas9 (Fig. 5F). Electron microscopy was then used to compare the morphology of mitochondria in M6 and the age-matched monkey brain (WT6) (Fig. 5G). Counting the percentage of different types of mitochondria (Type I: normal appearing cristae; Type II: swollen, irregular or whirling cristae; Type III: discontinuous outer membrane or deficient cristae; Type IV: both discontinuous outer membrane and swollen cristae) showed no difference in the striatum and substantia nigra between M6 and WT6 monkeys (Fig. 5H).

If *PINK1* mutations severely affect mitochondrial function, brain metabolomics may show alterations in the metabolites that reflect mitochondrial function. Since the 3-year-old monkey provided us with enough fresh brain cortical tissues, we used its tissues for metabolomics analysis but did not observe significant differences in most metabolites between M6 monkey and the age-matched wild type monkeys (Fig. S6A). This result was also supported by comparing the relative levels of metabolites such as creatine, phosphocreatine, ATP, high-energy phosphates (AMP, NADPH) and TCA cycle (citric acid), which are closely related to mitochondrial function, in the brain cortical tissues of M6 and WT monkeys (Fig. S6B).

### PINK1 deficiency reduces protein phosphorylation in the monkey brain

**Figure 6:**
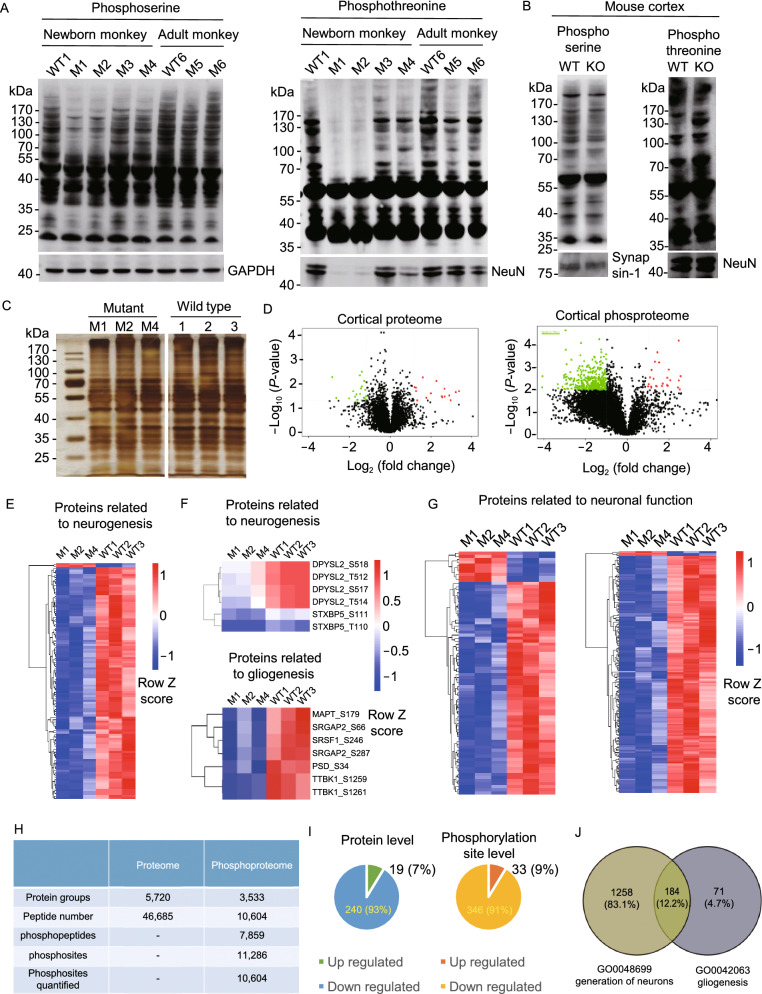
**Broad effects of PINK1 loss on protein phosphorylation**. (A) Western blot analysis of cortical lysates of *PINK1* mutant newborn (M1, M2, M3, M4) and adult (M5, M6) monkeys and one newborn (WT1) and one 3-year-old (WT6) wild-type monkeys. (B) Western blots of WT and *Pink1* knock-out (KO) mouse brain cortical tissues. In (A) and (B), the samples were probed with antibodies to phosphoserine, phosphotheronine, NeuN, synapsin-1, and GAPDH. (C) Silver staining of monkey brain cortical lysates (M1, M2, M4, and 3 WT newborn monkeys, 1, 2, 3) used for mass spectrometry. (D) Volcano plot of the monkey cortical proteome and phosphoproteome showing the upregulated (red spot) and downregulated (green spot) proteins between *PINK1* mutant and WT monkeys. X-axis represents the log2 ratio between mutant and WT, and the Y-axis represents log10 (*P*-value). Phosphopeptides that showed a 1.5-fold or greater change and p-value less than 0.05 were considered significantly up- or down-regulated. (E) Heatmap of the phosphorylation sites from proteins involved in neurogenesis. (F) Examples of protein whose expression is important for neurogenesis or gliogenesis and is also reduced in *PINK1* mutant monkey brains. (G) Heatmap of the phosphorylation sites from proteins involved in neuronal function. (H) The numbers of proteins, peptides, phosphopeptides, phophosites identified in monkey cortical tissues. (I) Summary of the up- and down-regulated proteins and phosphorylation sites in the *PINK1* mutant monkey cortices. The quantitative results were median-normalized followed by Student’s *t*-test assuming equal variance. (J) Summary of the down-regulated phosphorylation of proteins for neurogenesis and gliogenesis

Since PINK1 is a serine/threonine kinase, we next examined protein phosphorylation in PINK1-deficient monkey brain cortex using the same approach for cell cultures in our previous study (Wan et al., 2018). Our earlier study revealed that CRISPR/Cas9 mediated differing extents of *PINK1* deletion and neuronal loss in the brain cortical tissues of newborn monkeys (M1–M4) and adult monkeys (M5 and M6), with the greatest reduction of PINK1 in M1 and M2, a modest reduction in M3 and M4, and the least reduction in M5 and M6 (Yang et al., 2019b). These brain tissues had more homogenous PINK1 reduction and less variability in neuronal loss than viral-injected brain tissues and therefore were used for protein phosphorylation assays. Western blot analysis also revealed that the reduction in protein phosphorylation was most dramatic in M1 and M2, less remarkable in M3 and M4, and less noticeable in M5 and M6 (Fig. 6A). Consistent with the reduced protein phosphorylation, there was also a decrease in the neuronal protein NeuN. In *PINK1* KO mouse brains, however, there was no evident reduction of protein phosphorylation or neuronal proteins (synapsin-1, NeuN) (Fig. 6B).

We then performed mass spectrometry analysis of the proteomics and phosphoproteomics in cortical tissues available from *PINK1* mutant (M1, M2, and M4) and three WT age-matched postnatal monkeys (WT1-3). While the amounts of total proteins between WT and PINK1 mutant cortical tissues do not appear to be different, there was a significant reduction in the amount of phosphorylated proteins (Fig. 6C and 6D). Importantly, there was decrease in the phosphorylation of many proteins that are important for neurogenesis and neuronal function in *PINK1* mutant monkey brains (Fig. 6E–G). We analyzed a total of 46,685 peptides and 11,286 phosphorylation sites (Fig. 6H). The results demonstrated far more changes in phosphorylation reduction (91%) than up-regulation (9%) (Fig. 6I). Loss of PINK1 caused significant downregulation of phosphorylation of a number of proteins (83.15%) important for neurogenesis (Figs. 6J and S7). In contrast, fewer proteins (4.7%) related to gliogenesis showed a decrease in phosphorylation (Fig. 6J). Analysis of genes for immune response and endolysosomal sorting and trafficking only revealed that the phosphorylation of some endosomal function related proteins, which are also important for neuronal function, was reduced significantly in *PINK1* mutant monkey brain (Fig. S8A–C). We also compared phosphorylation profiling of mitochondria function related genes as well as early PD associated genes but found that their differences between WT and PINK1 mutant monkeys are limited and not obvious when compared with those for neuronal function (Fig. S8D and S8E). All these data collectively show that loss of PINK1 reduces the phosphorylation of various proteins that are important for neurogenesis and neuronal function.

### *PINK1* mutations affect protein phosphorylation in the monkey brain and human cells

**Figure 7:**
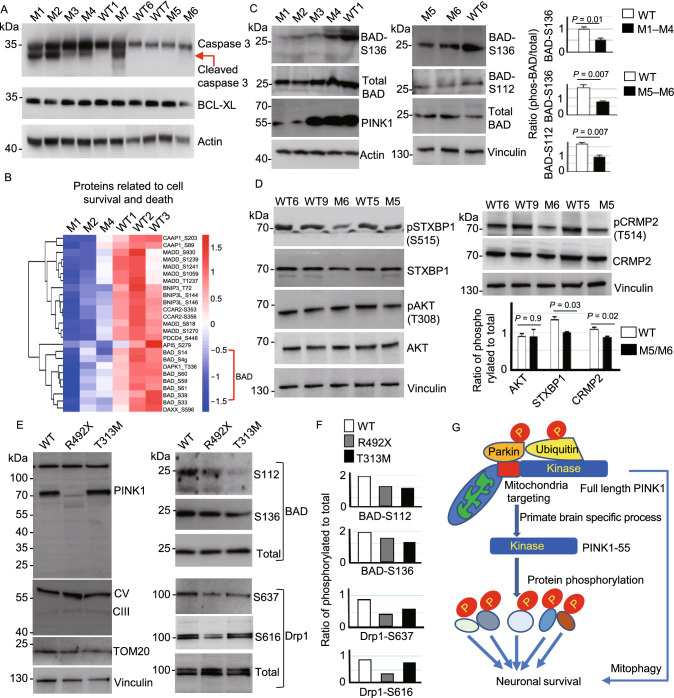
**PINK1 mutations affect protein phosphorylation**. (A) Western blot showing that caspase-3 cleavage occurred to different extents in different *PINK1* mutant monkeys as compared with WT monkeys. The brain cortical tissues were probed with antibodies to caspase-3, actin and BCL-XL. (B) Heatmap of the phosphorylation sites from proteins involved in neuronal apoptosis showing the reduced phosphorylation of the pro-apoptotic protein BAD. (C) Western blot using phospho-antibodies showing the decreased phosphorylation of BAD at S136 and S112 in *PINK1* mutant monkey brain cortex. The ratios of phosphorylated BAD to total BAD are presented in the right panel. (D) Western blot revealing decreased phosphorylation of neuronal proteins (STXBP1-S515, CRMP2-T514) but not Akt-T308. (E) Western blot analysis of wild type (WT), R492X, and T313M mutant human fibroblast cells after 10 μmol/L CCCP treatment for 12 h. Note that mitochondrial proteins (CIII, CV, and TOM20) were expressed at the similar levels in these human cells (left panel). The cell samples were probed with antibodies to BAD, Drp1, and their phosphorylated forms, and representative blot images are presented (right panel). (F) The ratios of phosphorylated BAD or Drp1 to the total BAD or Drp1 in (E). (G) A proposed model suggests that the kinase form of PINK1 (PINK1-55) is selectively expressed in the primate brain. Loss and mutations of PINK1 can affect its kinase activity and result in a broad reduction of protein phosphorylation, which can mediate neuronal loss independent of mitophagy that requires the targeting of full-length PINK1 to mitochondria

CRISPR/Cas9 targeting can cause various mutations, leading to different extents to which the targeted gene expression is reduced. We have shown that PINK1 is dramatically reduced in the brains of newborn M1 and M2 monkeys but is not significantly decreased in the newborn M3, M4, 1.5-year-old (M5), and 3-year-old (M6) monkey brains, perhaps because the in-frame or mosaic mutations rather than a large deletion did not significantly affect the level of PINK1 in M3–M6 monkeys (Yang et al., 2019b). Previous *in vitro* studies have shown that PINK1 phosphorylates Parkin at Ser65 and ubiquitin and mutations in PINK1 can affect its kinase activity (Lazarou et al., 2015; Ordureau et al., 2015; Gladkova et al., 2018; Wan et al., 2018; Han et al., 2020). Western blot revealed that loss of PINK1 in M1 and M2 could reduce the phosphorylation of Parkin-Ser 65 but PINK1 mutations in M3 and M4 did not (Fig. S9A). Similar to Parkin phosphorylation, ubiquitin phosphorylation was reduced in M1 and M2 monkey brains but did not show obvious reduction in M3 and M4 monkey brains (Fig. S9B), which was confirmed by the phosphate-affinity (Phos-tag) PAGE that can retard the migration of phosphorylated proteins to distinguish them from unphosphorylated proteins (Koyano et al., 2014) (Fig. S9C). It is clear that loss of PINK1 can induce a more dramatic and broad reduction of protein phosphorylation and affects cellular function to a greater extent than PINK1 mutations that do not decrease the level of PINK1. In support of this idea, Western blot analysis showed that caspase-3 cleavage, a process for apoptosis, was more pronounced in M1 and M2 than in other *PINK1* mutant monkey brains (Fig. 7A).

Since the majority of *PINK1* mutations in PD patients are point mutations that may not affect the level of PINK1 (Pickrell and Youle, 2015), it would be important to investigate the effect of *PINK1* mutations in those monkey brains that do not show significantly reduced expression of PINK1. By looking at phosphoproteomics data, we found that the phosphorylation of BAD, a proapoptotic member of the Bcl-2 family whose phosphorylation is important for neuronal survival (Datta et al., 2002), was also reduced in the M4 mutant monkey brain (Fig. 7B). We used a specific antibody to the phosphorylated BAD for Western blot and found that BAD phosphorylation at S112 and S136 was decreased in the cortical tissues of M3 and M4 monkey brains in which the PINK1 level was not significantly reduced (Fig. 7C). However, in the mouse brain, no obvious alteration of BAD phosphorylation was observed in the *Pink1* KO brain (Fig. S9D), again suggesting that endogenous Pink1 in the mouse brain is expressed at a very low level. In *PINK1* mutant monkey brains (M5 and M6), Western blot with available antibodies revealed selective phosphorylation reduction of CRMP2 and STXBP1, but not AKT (Fig. 7D), which confirmed mass spectrometry results and supported the idea that loss or mutations of PINK1 by CRISPR/Cas9 targeting can impair its kinase activity to reduce phosphorylation of some neuronal proteins.

If *PINK1* mutations affect protein phosphorylation, we may observe this defect in human cells expressing mutant PINK1. To test this idea, we examined fibroblast cells from patients with *PINK1* mutations (R492X and T313M) (Han et al., 2020). Because fibroblast cells express endogenous PINK1 at a very low level, we induced PINK1 expression by treating them with 10 µmol/L CCCP for 12 h and then analyzed the phosphorylation of BAD and Drp1, two substrates for PINK1 phosphorylation (Wan et al., 2018; Han et al., 2020). As R492X mutation leads to a truncated PINK1, R492X cells did not produce full-length PINK1 and showed a reduction in BAD and Drp1 phosphorylation without affecting the expression of mitochondrial proteins (CV, CIII, and TOM20) when compared with wild-type (WT) cells (Fig. 7E, left panel). Also, *PINK1* mutation (T313M) reduced the phosphorylation of BAD and Drp1, though the reduction of Drp1 phosphorylation was not great as that in R492X cells lacking the expression of full-length PINK1 (Fig. 7E and 7F). Based on these results, we propose that PINK1 (PINK1-55) is selectively expressed in the human and monkey brains to phosphorylate a large number of proteins. Loss of PINK1 can broadly affect phosphorylation of various proteins while mutations may more selectively affect phosphorylation of some proteins. Because of this unique and critical function of PINK1 in the primate brains, depletion of PINK1 in the monkey brains can lead to neuronal loss at different ages. Mutations in *PINK1* found in PD patients may also partially affect its kinase activity, and such kinase dysfunction can contribute to the late-onset neurodegeneration in Parkinson’s disease (Fig. 7G).

## DISCUSSION

Because of the lack of studies of the postmortem brains from patients with homozygous *PINK1* mutations, we do not know how *PINK1* mutations affect the brains of humans. However, our studies of the human and monkey tissues revealed several unexpected and important findings. First, PINK1 protein is selectively more abundant in the primate brains than the mouse brains. Second, acute loss of PINK1 can result in severe neurodegeneration in the adult monkey brains. Third, PINK1 dysfunction caused by CRISPR/Cas9 targeting did not alter mitochondrial morphology and dynamics. Finally, loss or mutations of PINK1 can reduce protein phosphorylation in the monkey brain and human cells. These interesting findings lead us to propose that the unique kinase function of PINK1 is important for neuronal survival in the primate brains and that its kinase dysfunction may also be involved in PD pathogenesis.

The higher expression level of PINK1 in human and monkey brains than rodent brains was clearly verified by comparing PINK1 in mice and monkeys using their PINK1 deficient models and six different antibodies. The selective expression of PINK1 in the primate brains is in clear contrast to the very low level of mouse Pink1 that could only be detected by immunoprecipitation (McWilliams et al., 2018). These findings have clarified some puzzling issues regarding *PINK1* knockout models of other species. Removal of Drosophila *Pink1* results in apoptotic muscle degeneration (Clark et al., 2006), which, however, does not mimic the selective neuronal loss in PD. On the other hand, in *Pink1* knockout mice (Kitada et al., 2007; Gispert et al., 2009; Xiong et al., 2009; Akundi et al., 2011) and pigs (Zhou et al., 2015; Wang et al., 2016), no obvious neurodegeneration nor severe phenotypes were reported. Although *Pink1* KO rats were found to have age-dependent loss of tyrosine hydroxylase (TH)-positive neurons (Dave et al., 2014), this mild degeneration is apparently different from the severe neuronal loss in *PINK1* mutant monkeys and could not be confirmed by other studies (de Haas et al., 2019). Intestinal bacterial pathogens were recently thought to promote neurodegeneration in *Pink1* knockout mice (Matheoud et al., 2019). However, the abundant expression of PINK1 and neuronal degeneration by directly targeting *PINK1* in the monkey brain indicate that neurodegeneration, at least in the primates, is primarily caused by neuronal PINK1 deficiency. Lack of robust neurodegeneration in the reported *PINK1* KO animals may be largely due to their intrinsic low level of PINK1 such that the undetectable or very low level of PINK1 is not essential for neuronal survival under normal physiological conditions.

Identification of the unique expression of PINK1 in the primate brains also provides new mechanistic insight into PD pathogenesis. *In vitro* studies and biochemical analysis have provided convincing evidence that PINK1 mediates mitophagy by its association with mitochondria and phosphorylation of Parkin and ubiquitin (Lazarou et al., 2015; Ordureau et al., 2015; Gladkova et al., 2018), leading to the prevalent theory that PINK1 mutations may impair mitophagy to cause PD neuropathology (de Vries and Przedborski, 2013; Pickrell and Youle, 2015; Chu, 2019). However, strong *in vivo* evidence for mitophagy involvement in neurodegeneration is still lacking (Whitworth and Pallanck, 2017; Cummins and Gotz, 2018). Instead, growing evidence demonstrates that the basal mitophagy function or mitochondrial function is not affected by the loss of PINK1 in *vivo* (Lee et al., 2018; McWilliams et al., 2018; Walsh et al., 2018). Our findings provide new evidence for the decreased phosphorylation of a number of proteins, including those needed for neurogenesis and neuronal function, in *PINK1* mutant monkey brains. This biochemical alteration is consistent with the unique expression of PINK1-55, a kinase form of PINK1, in the primate brains. The critical kinase function of PINK1 explains the severe neuronal loss in *PINK1* mutant monkeys and also highlights the possible involvement of its defect in patient brains with *PINK1* mutations.

If deletion of *PINK1* results in abnormal mitophagy in the brain as observed *in vitro*, it is expected to see the accumulation of damaged mitochondria, a reduced number of normal mitochondria, and altered levels of mitochondrial proteins in the CRISPR/Cas9 targeted monkeys. However, our EM examination did not reveal difference in mitochondrial morphology between *PINK1* mutant and WT monkey brains. Western blot of mitochondrial proteins did not show altered levels of mitochondrial marker proteins either in both *PINK1* mutant monkey brains and cultured primary neuronal cells, supporting the idea that the integrity of mitochondria was not altered in *PINK1* mutant monkey brains at early age. Despite these, it remains to be investigated whether PINK1 deficiency affects mitochondria activities, which can be measured by JC-1 or mitotracker dyes. Such experiment would require use of live brain cells from embryonic monkeys in the future study. In addition, we cannot rule out the possibility that PINK1 deficiency or mutation affects mitochondria and impacts mitophagy in the aged primate brains when mitochondria are damaged by age-related and cumulative cellular stress and insults. This is because the most common onset age for clinic symptoms of patients with *PINK1* mutations is between 30 and 40 years (Bentivoglio et al., 2001; Bonifati et al., 2005; Marongiu et al., 2008), though a homozygous missense mutation was found to cause the earliest onset parkinsonism in a 5-year-old boy (Al-Rumayyan et al., 2017). CRISPR/Cas9 mediated different types of PINK1 mutations in the non-human primates and caused different phenotypes (Yang et al., 2019a, b; Chen et al., 2021; Li et al., 2021). The differences in neurodegeneration and phenotypes between *PINK1* mutant monkeys and patients are very likely due to different types of *PINK1* mutations. The majority of *PINK1* mutations in humans are homozygous point mutations in the *PINK1* gene region encoding the kinase domain, with a few cases of heterozygous large deletions (Ishihara-Paul et al., 2008; Trinh and Farrer, 2013; Pickrell and Youle, 2015). Homozygous deletion of large fragments in the PINK1 gene has not been reported in humans, perhaps because such mutation is lethal to fetal development. The single locus mutations and heterozygous deletion may cause a partial loss of PINK1 expression or function, therefore leading to different ages of onset (ranging from 9 to 61 years) and varying degrees of phenotypes in patients. In contrast, CRISPR-mediated mutations, especially the large deletion resulting from targeting two different *PINK1* exons, can severely affect PINK1 expression and function, resulting in a more severe phenotype as seen in the recently reported monkey model (Yang et al., 2019b), in adult monkey brains and in primary cultured monkey neuronal cells in the current study.

Because phosphorylation of a number of proteins is an important regulatory mechanism for neuronal function and survival, the global defects of protein phosphorylation in *PINK1* mutant monkey brains are very likely to synergistically contribute to neuronal degeneration. For example, caspase-3 cleavage can enhance the pro-apoptotic effect of BAD (Condorelli et al., 2001). Thus, the severity of neurodegeneration is likely dependent on the extent to which PINK1 expression or function is affected. Given that PD is a global nervous system disorder with degeneration throughout the central nervous system (Braak et al., 2006; Langston, 2006), it is possible that PINK1 dysfunction affects various brain regions but neuronal cells in the substantia nigra may be more vulnerable, which could be related to the high level of PINK1 and its selective phosphorylation of cell-type specific proteins in this particular brain region.

Some studies have raised the possibility that PINK1’s function may be beyond mitophagy (Scarffe et al., 2014; Voigt et al., 2016). Also, PINK1 dysfunction is thought to be involved in cancers and other diseases in humans (O’Flanagan and O’Neill, 2014; Arena and Valente, 2017). Our findings demonstrated for the first time that PINK1 kinase is selectively expressed in the primates, suggesting that PINK1 kinase activity, rather than its mitophagy function, may also be involved in other human diseases. Although further study is required to understand how PINK1 kinase form is uniquely and selectively expressed in primate brain tissues, the current study using non-human primates provides new evidence for the *in vivo* function of PINK1 and pave a new avenue to further investigate PD pathogenesis and other human diseases that are also associated with PINK1 dysfunction.

## MATERIALS AND METHODS

### Monkeys

Generation of PINK1 mutant rhesus monkeys via embryonic injection of CRISPR/Cas9 was described in our early studies (Yang et al., 2019b) using the same gene targeting approach on monkey embryos described in our previous studies (Yang et al., 2015; Tu et al., 2017; Zhao et al., 2017; Yang et al., 2019b). During our previous studies, *in vitro* fertilization of the injected embryos and embryo transfer had yielded a number of aborted fetuses or stillborn monkeys that carried mutations in specific genes or were wild type without any gene targeting. The brain tissues isolated from wild type and gene-targeted rhesus monkeys were saved and used in the current studies. Information of monkeys used for the study is listed in Table S1. For viral injection of rhesus monkey brains, we used the method described in our previous studies (Yang et al., 2015). The monkeys were housed at Yuanxi Biotech Inc. Guangzhou. All animal procedures were approved by the Institutional Animal Care and Use Committee at Yuanxi Biotech Inc. Guangzhou. This study occurred in strict compliance with the “Guide for the Care and Use of Laboratory Animals of the Institute of Laboratory Animal Science (est. 2006)” and “The use of non-human primates in research of the Institute of Laboratory Animal Science (est. 2006)” to ensure the safety of personnel and animal welfare.

### Human tissues

The use of postmortem human brains was approved by the Ethics Committee of Central South University Xiangya School of Medicine, in compliance with the Code of Ethics of the World Medical Association (Declaration of Helsinki). Postmortem peripheral tissues and brains were collected through the willed body donation program within 24 h after death. The postmortem tissues used were from two individuals who died of paraplegia (54-year-old, man) and lymphoma (51-year-old, man) with postmortem interval 5 h and 9 h, respectively. The postmortem brains were assessed for optimal histological integrity following a standard protocol proposed by the China Human Brain Banking Consortium (Yan et al., 2015; Qiu et al., 2019).

### Mice

*Pink1* knockout mice were generated and bred by Beijing Biocytogen, China. Briefly, gRNAs were designed to target exon 2 and exon 4 in the mouse *Pink1* gene. Cas9 mRNAs and gRNAs were mixed for pronuclear injection into the mouse zygotes (C57BL/6). The mice of generation F0 were genotyped with the following PCR primers: sense 5′-CTCCCCACTCTTGTGTTTGCTATGT-3, and antisense 5′-CAGTTGCTGCTCAGAGTAGTTCACA-3′ for exon 2 genotyping; sense 5′-CACCATGTGAGATGGATAGATGGGC-3′ and antisense 5′-AAGTTAGCTGGCACTGAAAGAGGAC-3′ for exon 4 genotyping. Mice carrying *Pink1* mutations were used to breed to generate F1 and F2 generation mice. The mice carrying the mutations in both exon 2 and exon 4 in two alleles of *Pink1* were treated as *Pink1* knockout mice and used with the wild type littermates for further examination.

### Reagents

Antibodies and reagents used in the current study are listed in Table S2.

### Western blot analysis, immunohistochemistry, and electron microscopy

For western blot analysis, monkey brain tissues were lysed in ice-cold RIPA buffer (50 mmol/L Tris, pH 8.0, 150 mmol/L NaCl, 1 mmol/L EDTA pH 8.0, 1 mmol/L EGTA pH 8.0, 0.1% SDS, 0.5% DOC, 50 mmol/L NaF and 1% Triton X-100) containing Halt protease inhibitor cocktail (Thermo Scientific) and PMSF. The lysates were incubated on ice for 30 min, sonicated, and centrifuged at the maximum speed for 10 min. Equal amounts of proteins from the supernatants determined by BCA assay were resolved by SDS-PAGE and subjected to Western blot analysis with appropriate primary antibodies (See Table S2). Acquired images were subjected to densitometric quantitation using ImageJ software.

For immunohistochemistry, monkey brain tissues were fixed overnight (12–16 h) in 4% PFA (paraformaldehyde) in 0.01 mol/L PBS, and then transferred into 30% sucrose at 4 °C to let the brain completely sink to the bottom of the tube. Brain tissue was sectioned at 20 μm using a cryostat at −19 °C. Monkey tissue slides were fixed for 10 min in 4% PFA in 0.01 mol/L PBS at room temperature, blocked with 0.1% Triton X-100/2% NGS /3% BSA/1× PBS for 30 min, and incubated with primary antibodies to relative proteins in 3% BSA/2% NGS/1× PBS overnight at 4 °C. The slices were washed three times with 1× PBS and rinsed with secondary antibodies. Double immunofluorescence staining was analyzed using a confocal imaging system ((Olympus FV3000 Microscope).

For electron microscopy (EM), *PINK1* mutant (M6) monkey was deeply anesthetized by intraperitoneal injection of 0.3–0.5 mL of atropine, followed by 10–12 mg of ketamine and 15–20 mg of pelltobarbitalum natricum per kg body weight. The freshly isolated brain tissues from sacrificed M6 and the age-matched monkey were fixed with 2.5% glutaraldehyde/0.1 mol/L PB overnight at 4 °C. Monkey brains were sectioned into 50 μm using a vibratome (Leica, VT1000s) and the sections were processed for electron microscopic examination. In brief, all sections were osmicated in 1% OsO4 in 0.1 mol/L PB and embedded in Eponate12 (Ted Pella). The dried brain sections were cut into ultrathin sections (60 nm) with a Leica Ultracut S ultramicrotome under a Hitachi H-7500 transmission electron microscope equipped with a Gatan Bio-Scan CCD camera.

### AAV viral preparation and stereotaxic injection

CRISPR/Cas9-expressing viral vectors (PX551, PX552) were obtained from Addgene (plasmids #60957 and 60958). AAV-PINK1-gRNAs were generated by inserting gRNAs into PX552 via SapI restriction sites. gRNA sequences are as follows: *PINK1* exon 2 sgRNA: 5′-GGCTGGAGGAGTATCTGATAggg-3′, *PINK1* exon 4 sgRNA: 5′-ccgGGTTCTCCGCGCTTTCACC-3′, and control gRNA: ACCGGAAGAGCGACCTCTTCT (PAM sequences are shown in lowercase). AAV-CMV-Cas9 vector was generated by replacing Mecp2 promoter (228 bp) in PX551 with CMV promoter (658 bp) using *Xba*I and *Age*I restriction sites. These viral vectors were packaged to generate purified AAV9 viruses. The genomic titer of viruses (vg) (approximate 10^12^ vg/mL) of the purified viruses, which were used for injection, was determined by PCR method.

Viral injection of monkey brains was performed using the method described in our previous studies (Yang et al., 2015). We used four wild type rhesus monkeys (one 3-year-old, two 12-year-old and one 10-year-old). The monkey brain regions were located by MRI before injection. Each monkey was anesthetized by intraperitoneal injection of 0.3–0.5 mL of atropine, followed by 10–12 mg of ketamine and 15–20 mg of pelltobarbitalum natricum per kg body weight. The monkeys were then stabilized on a stereotaxic instrument (David Kopf Instruments). Five to ten microliters of viruses were injected into one side of the monkey prefrontal cortex, striatum, or substantia nigra. The right side of these brain regions were injected with mixed AAV-Cas9 with AAV-PINK1 gRNA (1:4 ratio). The left side was injected with control AAV-GFP or AAV-HTT gRNA or AAV-Control gRNA. After injection 2–3 months, the monkeys were euthanized by deep anesthesia with intraperitoneal injection of 0.3–0.5 mL of atropine, followed by 10–12 mg of ketamine and 15–20 mg of pelltobarbitalum natricum per kg body weight. The brain tissues of the monkeys were then isolated for immunohistochemical analysis.

### Phos-tag PAGE

To examine the *in vivo* phosphorylation levels of ubiquitin of wild type or PINK1 mutant monkey brains, phos-tag SDS-PAGE was made using phos-tagTM acrylamide from Wako Pure Chemical Industries, Ltd. (Osaka, Japan) by following the instruction of the manufacturer. Briefly, monkey brain cortical tissues were homogenized with RIPA buffer containing EDTA-free protease inhibitor cocktail supplemented with phos-stop (Roche). Proteins were resolved by 12% SDS-PAGE using a gel containing 50 μmol/L phos-tag acrylamide and MnCl_2_ at 85 V. Before blot transfer, gels were incubated twice in SDS-PAGE transfer buffer containing 10 mmol/L EDTA for 20 min. Gels were then incubated in SDS-PAGE transfer buffer without EDTA for 10 min. Gel transfer and immunoblotting were performed by following a standard procedure.

### Culture of monkey primary cortical neurons

Cortical tissues were isolated from a fetal monkey brain at embryonic day 90. Dissected tissues were treated with 0.0625 mg/mL trypsin in 1× HBSS buffer without calcium or magnesium for 10 min at 37 °C, followed by triturating with a 1 mL pipette tip 20 times. Cells were then washed once with the tissue culture medium and centrifuged at 250 ×*g* for 5 min. Cells were plated on glass coverslips that had been precoated with 0.1 mg/mL poly-D-lysine and 1 g/mL laminin, and grown in neurobasal/B27 medium at a density of 10^5^/mL. Transfection of cultured neurons via electroporation was performed using Nucleofector Kit (Lonza) before plating primary neurons on poly-D-lysine coated 12-well plates. In brief, 1 × 10^6^ cells were used for electroporation with 3 μg plasmids (control gRNA-RFP/Cas9 or PINK1 gRNA-RFP/Cas9). The cultured cells at 14 d *in vitro* (DIV) were collected for Western blot or immunostaining.

### Human fibroblasts culture and treatment

Human fibroblasts from PD patients with PINK1 mutations (R492X and T313M) and control cells from a normal individual were obtained and cultured as described previously (Han et al, 2020). Briefly, the cells were cultured in Dulbecco’s modified Eagle’s medium (Thermo Fisher) supplemented with 10% fetal bovine serum and 1% penicillin-streptomycin. All fibroblasts had the same passage number for CCCP (Sigma) treatment (10 µmol/L for 12 h) and Western blot. Passage numbers < 10 were used for all experiments.

### Mitochondrial and cytosolic fractionation

The fractionation of mitochondria and cytoplasm was performed as described previously (Clayton and Shadel, 2014). Briefly, 20 mg monkey brain cortical tissues were homogenized in 1 mL ice-cold lysis buffer (210 mmol/L mannitol, 70 mmol/L sucrose, 5 mmol/L Tris-HCl, pH 7.5, 1 mmol/L EDTA, pH 7.5) containing 1 mmol/L DTT and protease inhibitors. The homogenates were centrifuged at 700 ×*g* for 10 min at 4 °C to remove nuclei, unbroken cells, and large membrane fragments. The supernatants were transferred to a clean tube to pellet the mitochondria materials and isolate the cytosolic fraction at 10,000 ×*g* for 30 min at 4 °C. The pellets were washed with lysis buffer and centrifuged again to precipitate mitochondria at 17,000 ×*g* for 15 min. For Western blot, 10 μg each of the cytosolic and mitochondrial fractions were loaded on a 12% SDS-gel.

### Mitochondrial dynamic analysis

The primary cultured monkey astrocytes mitochondrial system was assessed 14 d after electroporation. In brief, 1 × 10^6^ cells were used for electroporation with 3 μg plasmids (control gRNA-RFP/Cas9 or PINK1 gRNA-RFP/Cas9). To label mitochondria, cells were incubated with MitoTracker probes (M7514, invitrogen) as the instruction of the manufacturer described. Living cells were then imaged by acquisition of multiple optical sections with Olympus Spin SR confocal system. For mitochondrial number, single mitochondria were manually counted from live imaging acquisition based on merged green and red channels. Mitochondria length was manually calculated with OLYMPUS cellSens Dimension software (>1000 mitochondria were counted for each group). 15–20 RFP positive cells were used to quantify the number, length and motility of mitochondria for each group. Time-lapse images of mitochondrial movement were recorded using the 100× objective for a total duration of 5 min (every 3 second per frame). The dynamic changes in mitochondria size were assessed by measuring three subtypes of mitochondria based on the intact length: (>2.5 μm), fragmented length (< 1 μm), and intermediate pattern (1.0–2. 5 μm).

To assess motility of mitochondria, we manually traced the position and lengths of individual mitochondria using the live time-series imaging within MATLAB. Considering the fluorescence quenching with longer imaging time, we traced the mitochondria movement for the first 90 s to avoid tracing inaccuracy. The total track length of mitochondria was calculated based on the tracing positions.

### Quantitative proteomics and phosphoproteomics

Monkey brains were lysed in urea buffer (7 mol/L urea, 2 mol/L thiourea, 50 mmol/L Tris-HCl, 150 mmol/L NaCl, 1 mmol/L EDTA, pH 7.5, with protease and phosphatase inhibitors). For TMT labeling, 330 μg protein lysate per sample were reduced with 10 mmol/L DTT at 55 °C for 30 min, alkylated with 50 mmol/L iodoacetamide in the dark for 30 min, precipitated with six volumes of pre-chilled acetone overnight, and digested with trypsin (1:100, Promega) overnight at 37 °C in 100 mmol/L TEAB. Peptides from the six conditions were labeled with 0.8 mg of TMT sixplex (Thermo Scientific, #90066) respectively, and combined afterward following the manufacturer’s instructions.

A total of 1.98 mg tryptic peptides were separated into 60 fractions using high-pH RPLC (Waters XBridge C18 column 5 μmo/L, 250 mm × 4.6 mm; mobile phase A (5 mmol/L NH_4_COOH, 2% acetonitrile, pH = 10.0) and B (5 mmol/L NH_4_COOH, 98% acetonitrile, pH = 10.0) at a flow rate of 1 mL/min using the following linear gradient: 0–6% phase B for 6 min, 6%–28.5% phase B for 30 min, 28.5%–34% phase B for 5 min, 34%–60% phase B for 10 min, 60% phase B for 9 min. The eluate was collected 1 min into vials (1 mL/tube). Finally, 60 fractions were combined into 12 components in zigzag fashion.

We took 5% of each fraction for the proteome analysis. The rest of the proteins were vacuum dried for phosphoproteome analysis. Samples were further enriched for phosphopeptides with titanium dioxide beads (TiO_2_, 5 μmo/L titansphere, GL Sciences Japan). TiO_2_ beads were pre-incubated in 2,5-dihydroxybenzoic acid (20 mg/mL) in 80% acetonitrile (ACN) and 0.5% acetic acid (DHB solution) for 20 min. Prior to addition of the beads, the peptide was brought to 80% acetonitrile, 1% trifluoroacetic acid (TFA) and 0.2 g/mL phthalic acid. TiO_2_ beads (0.13 milligram in 4 μL of DHB solution) were added to each sample and incubated at room temperature for 15 min while rotating, and the supernatant was then removed to a new tube for a repeat enrichment. The beads were combined in 100 μL wash buffer 1 (5 mmol/L KH_2_PO_4_, 30% ACN, 350 mmol/L KCl), followed by 100 μL wash buffer 2 (40% ACN, 0.5% acetic acid, 0.05% TFA) and 200 μL wash buffer 3 (80% ACN, 0.5% acetic acid). The phosphorylated peptides were then eluted first with 40 μL of 5% NH_4_OH followed by 40 μL of 10% NH_4_OH with 25% ACN. Eluted peptides were concentrated in a speed Vac, loaded onto C18 STAGE-tips and primed for LC−MS analysis.

Peptides were analyzed on an EASY-nLC1000 LC (Thermo Scientific) coupled on line with the Q-Exactive Orbitrap mass spectrometer (Thermo Scientific). Peptides were separated on an in-house packed C18-column (15 cm, 75 μmo/L I.D., 3 μmo/L p.s.) with a 120-min gradient from 12% to 32% buffer B (98% ACN, 0.1% formic acid) at a flow rate of 250 nl/min. One full scan MS from 400 to 1,600 m/z followed by 12 MS/MS scan were continuously acquired. The resolution for MS was set to 70,000 and for data-dependent MS/MS was set to 35,000. The nano-electrospray source conditions were: spray voltage, 1.8 kV; no sheath and auxiliary gas flow; heated capillary temperature, 275 °C. For HCD, the isolation window was set to 2 m/z and the normalized collision energy of 32 was applied.

### Metabolites identification and quantification

Monkey brain cortical tissues (100 mg) were used for HPLC-MS/MS Analysis of Metabolites by Novogene Corporation (ExionLC™ AD system (SCIEX) coupled with a QTRAP® 6500+ mass spectrometer (SCIEX)). The detection of the experimental samples using MRM (Multiple Reaction Monitoring) was based on Novogene in-house database. The Q1, Q3, RT (retention time), DP (declustering potential), and CE (collision energy) were used for the metabolite identification. The data files generated by HPLC-MS/MS were processed using the SCIEX OS Version 1.4 to integrate and correct the peak. The main parameters were set as follows: minimum peak height, 500; signal/noise ratio, 10; gaussian smooth width, 3. The area of each peak represents the relative content of the corresponding substance.

### Analysis of mass spectrometry data

The MaxQuant (version 1.5.3.30) software was used to analyze the MS/MS raw data. For the database searching parameters, the precursor mass tolerance was set to 15 ppm. Trypsin/P was set as the protease, accounting for in-source fragmentation of lysine or arginine residues followed by proline. Two missed cleavages were allowed. All data were searching against with the UniPort Macaca mulatta database (sequences) including oxidation (+15.9949 Daltons) of methionine and phosphorylation (+79.9663 Daltons) of serine, threonine, and tyrosine as dynamic modifications, and TMT ten plex (+229.1629 Daltons) on lysine and peptide N termini and carbamidomethylation (+57.0215 Daltons) of cysteine as static modifications.

### Analysis of metabolites data

The metabolites were annotated using the KEGG database (http://www.genome.jp/kegg/), HMDB database (http://www.hmdb.ca/) and Lipidmaps database (http://www.lipid maps.org/). The metabolites with VIP > 1 and *P*-value < 0.05 and fold change ≥ 2 or FC ≤ 0.5 were considered to be differential metabolites. Volcano plots were used to filter metabolites of interest, based on Log2(FC) and -log10(*P*-value) of metabolites. For clustering heat maps, the data were normalized using z-scores of the intensity areas of differential metabolites and were plotted by heatmap package in R language.

### Statistical analysis

Statistical significance was assessed using the 2-tailed Student’s *t*-test for comparing two groups. When analyzing multiple groups, we used one-way ANOVA to determine statistical significance. For monkeys that were repeatedly subjected to behavioral tests, we analyzed the data using two-way ANOVA. Data presented in figures are the mean ± SEM. Calculations were performed with GraphPad Prism software.

## Supplementary Information

Below is the link to the electronic supplementary material.

Supplementary file1 (PDF 2692 kb)

Supplementary file2 (MP4 923 kb)

Supplementary file3 (MP4 688 kb)

Supplementary file4 (MP4 664 kb)

## Data Availability

The data from this study will be made available upon request. The mass spectrometry data was deposited on ProteomeXchange with identifier PXD023520.

## DECLARATIONS

This work was supported by Key Field Research and Development Program of Guangdong province (2018B030337001), The National Natural Science Foundation of China (32070534, 81830032, 31872779, 82071421, 81873736, 82071223); Guangzhou Key Research Program on Brain Science (202007030008), The National Key Research and Development Program of China Stem Cell and Translational Research (2017YFA0105102, 2017YFA0105201), Guangdong Introducing Innovative and Entrepreneurial Teams award (2021ZT09Y007), Guangdong Key Laboratory of non-human primate research (No. 2020B121201006), Fundamental Research Funds for the Central Universities (Jinan University, 21620358), Yuanxi Biotech Inc. Guangzhou, and Hubei Topgene Biotechnology Co. ltd.

All animal procedures were approved by the Institutional Animal Care and Use Committee at Yuanxi Biotech Inc. Guangzhou.

Informed consent was obtained from all individual participants included in the study. Authors are responsible for correctness of the statements provided in the manuscript.

All authors give their consent for the publication of this study.

The datasets generated during and/or analyzed during the current study are available from the corresponding author on reasonable request.

W.Y., S.L., and X-J. L. designed the research experiments. W.Y., X.G., Z.T., X.C., R.H., Y. L., S.Y., X. Z., Y. Z., H.Y., Y.P., Q. W., Z.W., and X.X.C. performed the experiments. H.W., Y.Z., and L.L. performed mass spectrometry, J.G provided human cells, and X.Y. provided human postmortem tissues. W.Y. and X-J.L. wrote the manuscript. S.L. edited the manuscript.
