# Supplementary material for: PINK1 kinase dysfunction triggers neurodegeneration in the primate brain without impacting mitochondrial homeostasis
Source: Protein Cell. 2021 Nov 20;13(1):26–46. doi: 10.1007/s13238-021-00888-x (PMC8776976; doi:10.1007/s13238-021-00888-x)
Supplement: Supplementary file 1 — Supplementary file1 (PDF 2692 kb) [file 13238_2021_888_MOESM1_ESM.pdf]

Supplementary Information

PINK1 kinase dysfunction triggers neurodegeneration in the primate brain without impacting mitochondrial homeostasis

Weili Yang et al.,

Supplementary video 1

The walking and movement of a 3-year-old monkey showing abnormal and difficult movement of left limbs that are controlled by the right side of the substantia nigra, which was injected with AAV-PINK1-gRNA/Cas9. The movement of the right limbs is controlled by the left side of the substantia nigra injected with the control AAV-GFP. The video was recorded 2 months after injection.

Supplementary video 2

A representative live imaging video of primary cultured monkey glia cell transfected with AAV-Control-gRNA/Cas9 at day 14.

Supplementary video 3

A representative live imaging video of primary cultured monkey glia cell transfected with AAV-PINK1-gRNA/Cas9 at day 14.

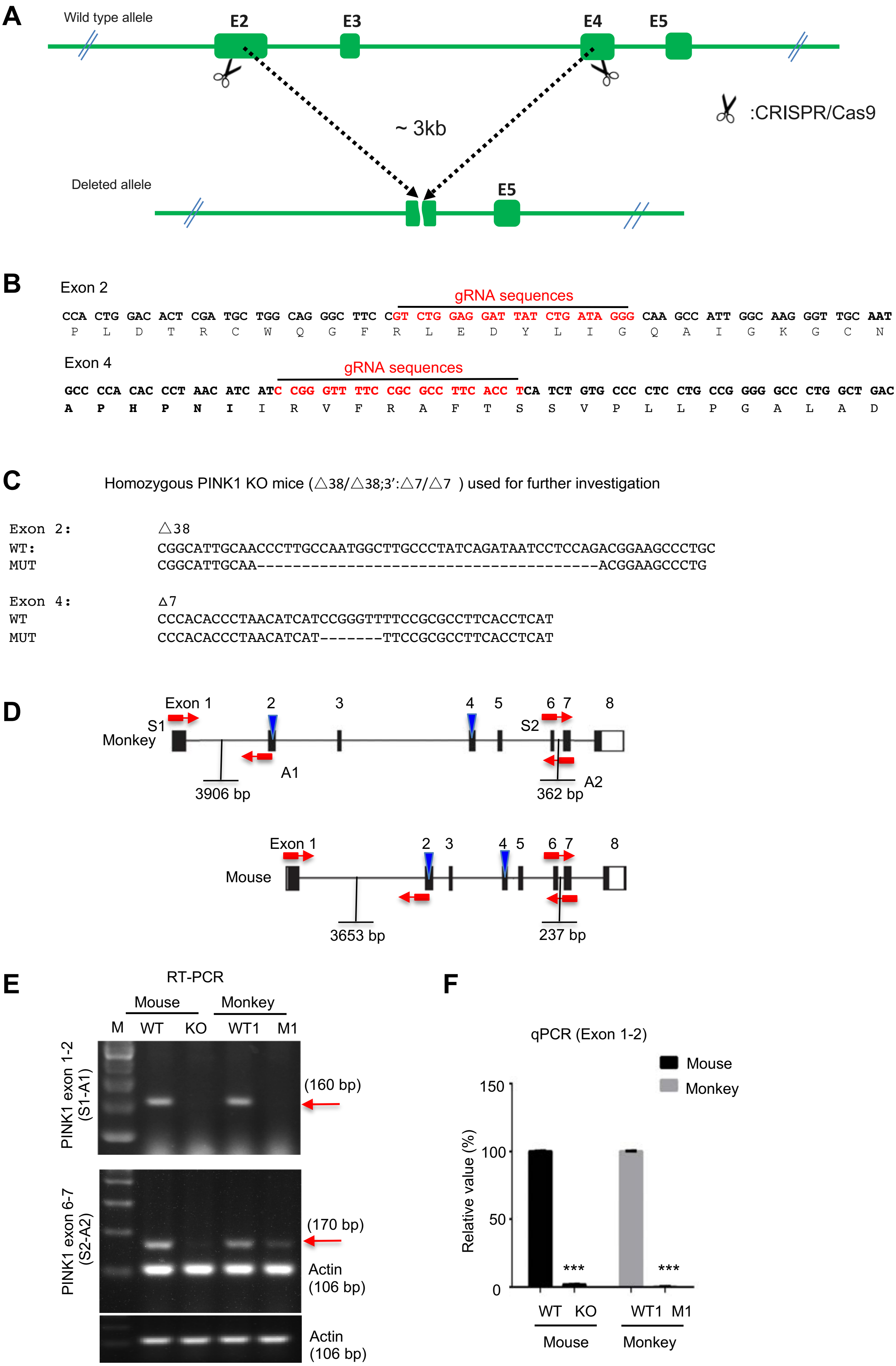

A
Wild type allele
E2
E3
E4
E5
:CRISPR/Cas9
~ 3kb
Deleted allele
E5
B
Exon 2
gRNA sequences
CCA CTG GAC ACT CGA TGC TGG CAG GGC TTC CGT CTG GAG GAT TAT CTG ATA GGG CAA GCC ATT GGC AAG GGT TGC AAT
P L D T R C W Q G F R L E D Y L I G Q A I G K G C N
Exon 4
gRNA sequences
GCC CCA CAC CCT AAC ATC ATC CGG GTT TTC CGC GCC TTC ACC TCA TCT GTG CCC CTC CTG CCG GGG GCC CTG GCT GAC
A P H P N I I R V F R A F T S S V P L L P G A L A D
C
Homozygous PINK1 KO mice (△38/△38;3':△7/△7) used for further investigation
Exon 2: △38
WT: CGGCATTGCAACCCTTGCCAATGGCTTGCCCTATCAGATAATCCTCCAGACGGAAGCCCTGC
MUT CGGCATTGCAA--------------------------------------ACGGAAGCCCTG
Exon 4: Δ7
WT CCCACACCCTAACATCATCCGGGTTTTCCGCGCCTTCACCTCAT
MUT CCCACACCCTAACATCAT-------TTCCGCGCCTTCACCTCAT
D
Exon 1
2
3
4
5
6 7
8
S1
S2
Monkey
A1
A2
3906 bp
362 bp
Exon 1
2 3
4 5
6 7
8
Mouse
3653 bp
237 bp
E
RT-PCR
Mouse
Monkey
M WT KO WT1 M1
PINK1 exon 1-2 (S1-A1)
(160 bp)
PINK1 exon 6-7 (S2-A2)
(170 bp)
Actin (106 bp)
Actin (106 bp)
F
qPCR (Exon 1-2)
Mouse
Monkey
Relative value (%)
150
100
50
0
***
***
WT KO
WT1 M1
Mouse
Monkey

Fig. S1. Generation of Pink1 KO mice by targeting exon 2 and exon 4 via CRISPR/Cas9.

(**A**) Exon 2 and exon 4 of the mouse *Pink1* gene were targeted by CRISPR/Cas9. (**B**) Sequences of gRNAs used for targeting exon 2 and exon 4 of the mouse *Pink1* gene. (**C**) DNA sequences of the targeted exon 2 (△38/△38) and exon 4 (△7/△7) in homozygous *Pink1* KO mice (△38/△38; △7/△7). (**D**) Two pairs of RT-PCR primers (S1 and A1; S2 and A2) were designed to detect *PINK1* transcripts of exon 1-2 and exon 6-7, respectively, in monkeys and mice. (**E**) Total RNA isolated from monkey and mouse cortical tissues was used to synthesize cDNA with oligo d(T)18 primers. RT-PCR were performed using primers indicated in (A), and the expected transcript products are indicated by arrows. PINK1 transcript expression was not detected in homozygous *Pink1* KO mouse and was reduced significantly in M1 monkey when compared with wild type (WT) animals. (**F**) Quantitative assessment of the relative levels of *PINK1* mRNA expression (% of actin) by qPCR (n= 3 experiments, 3 WT and 3 *Pink1* KO mice were examined for qPCR). *** $p<0.001$.

A

Sequence comparison of the PINK1 epitope for anti-N-terminal PINK1 (BC100-494, S086D, S085D) in different species

Human 175-mptlpq nlevtkstgl lpgrgpgtsa pgegqerapg apafplaikm mwnisagsss eailntmsqe lvpasrvala-250 BC100-494; S085D
Monkey 175-mpalpq nlevtkstgs lpgrgpgtsa pgeeqeqalg apafplaikm mwnisagsss eailntmsqe lvpasrvala-250
Mouse 175-mptlpq hlekakhlgl i-gkgpdvvl kgadgeqapg tptfpfaikm mwnisagsss eailskmsqe lvpasrvala-252 S086D

Sequence comparison of the PINK1 epitope for anti-C-terminal PINK1 (Ab23707) in different species

```
Human  484-lvrall qreaskrpsa rvaan-504 Ab23707
Monkey 484-lvrall qreaskrpsa rvaan-504
Mouse  484-lvrall qreaskrpsa rlaan-504
```

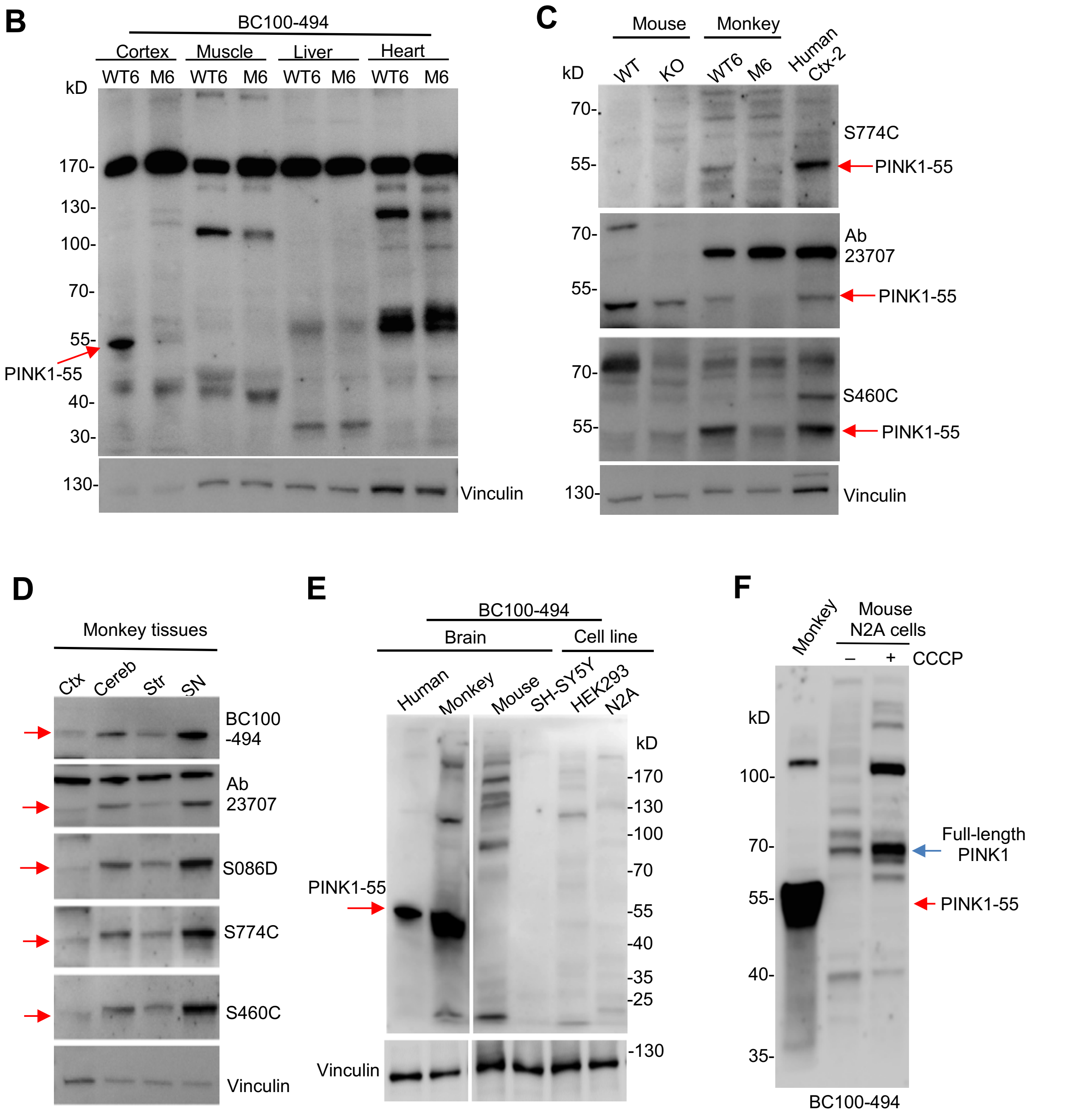

Fig. S2. Antibodies used to detect PINK1. (**A**) PINK1 amino acid sequence in different species and comparison of the epitope regions for anti-PINK1 antibodies. Amino acids in red are those that are different from human PINK1. **(B)** BC100-494 Western blotting of brain cortical and peripheral tissues of wild type (WT6) and *PINK1* mutant (M6) monkeys. The results show selective reduction of PINK1-55 in M6 and no differences in the peripheral tissues between WT6 and M6, indicating that PINK1-55 is selectively expressed in the monkey brain. (**C**) Three different antibodies (S774C, Ab23707, S460C) all reacted with PINK1-55 in wild type monkey cortex (WT6) and human brain cortex (Ctx-2). (**D**) PINK1-55 is more abundant in the monkey substantia nigra (SN) than in other brain regions (cortex: Ctx; cerebellum: Cereb; striatum: Str; brain stem: BS). The same monkey brain tissues were probed with five different antibodies and anti-vinculin. (**E**) Western blot analysis of brain tissue lysates of human, monkey, mouse, and human (SH-SY5Y, HEK293) or mouse (N2A) cell lines. BC100-494 only detected PINK1-55 (arrow) in the primate brains. Human brain hippocampus and monkey brain cortex were used. (**F**) BC100-494 was able to recognize the full-length PINK1 in mouse N2A cells treated with 10 μM CCCP for 12 h.

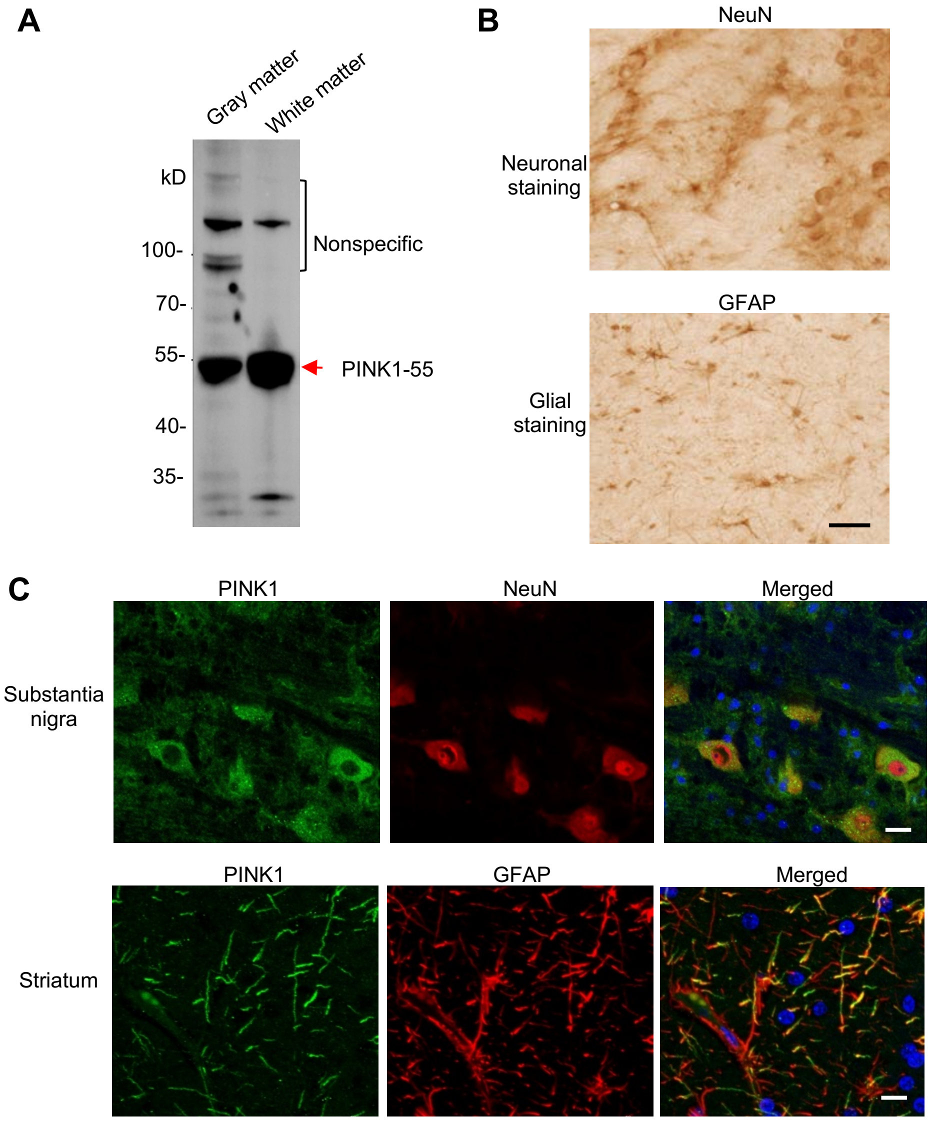

Fig. S3. PINK1 is expressed in neurons and astrocytes in the monkey brain. (**A**) BC100-494 Western blotting showing that PINK1-55 is expressed in the gray matter and white matter in the wild-type (WT6) monkey brain. (**B**) Representative immunostaining images of the brain stem of the wild-type monkey (WT6) stained by anti-PINK1 (BC100-494). PINK1 is distributed in the bodies and processes of neuronal and glial cells. Scale bars: 20 μm. (**C**) Double immunofluorescent staining of the substantia nigra (upper panel) and striatum (low panel) showing PINK1 (green) is expressed in neuronal (NeuN) and astrocytes (GFAP) in the monkey brain. Scale bars: 20 μm in the upper panel and 10 μm in lower panel.

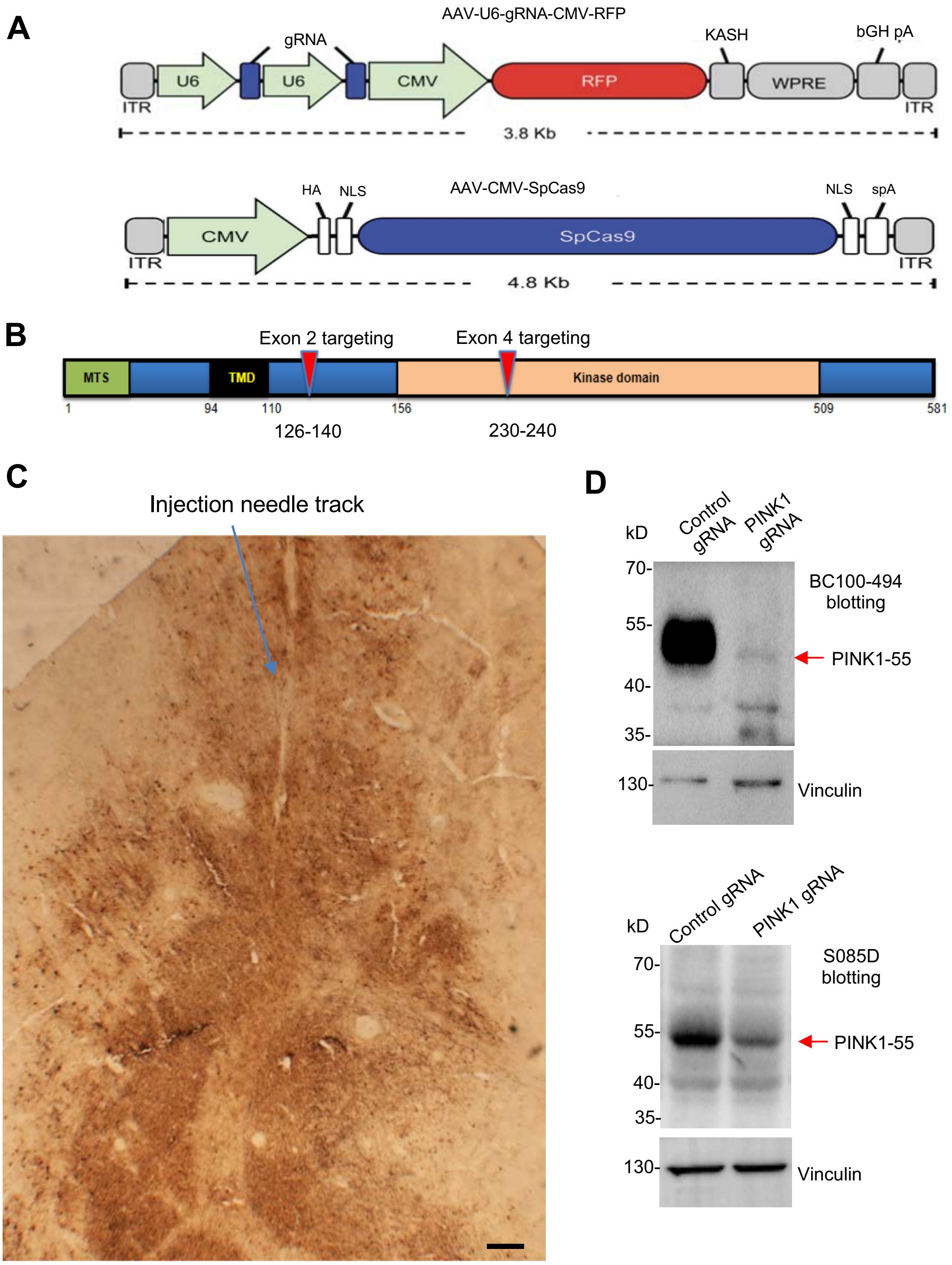

Fig. S4. AAV vectors used for expressing Cas9 or gRNA and RFP in the monkey brain. (**A**) A schematic representation of AAV9-PINK1-gRNA and AAV9-CMV-Cas9 constructs that were used for generation of AAV viruses for stereotaxic injection into the monkey brain. (**B**) PINK1 protein domains and targeted regions. MTS: a mitochondrial-targeting sequence; TMD: transmembrane domain; exon 2 and exon 4 targeting regions. (**C**) RFP immunostaining of AAV-injected monkey brain (prefrontal cortex) shows abundant RFP signals along the injection needle track. Expression of RFP was detected via immunohistochemistry with anti-RFP. Scale bar: 100 µm. (**D**) Western blot analysis of AAV-injected monkey brain substantia nigra using two antibodies (BC100-494 and S085D) showed obvious reduction of PINK1.

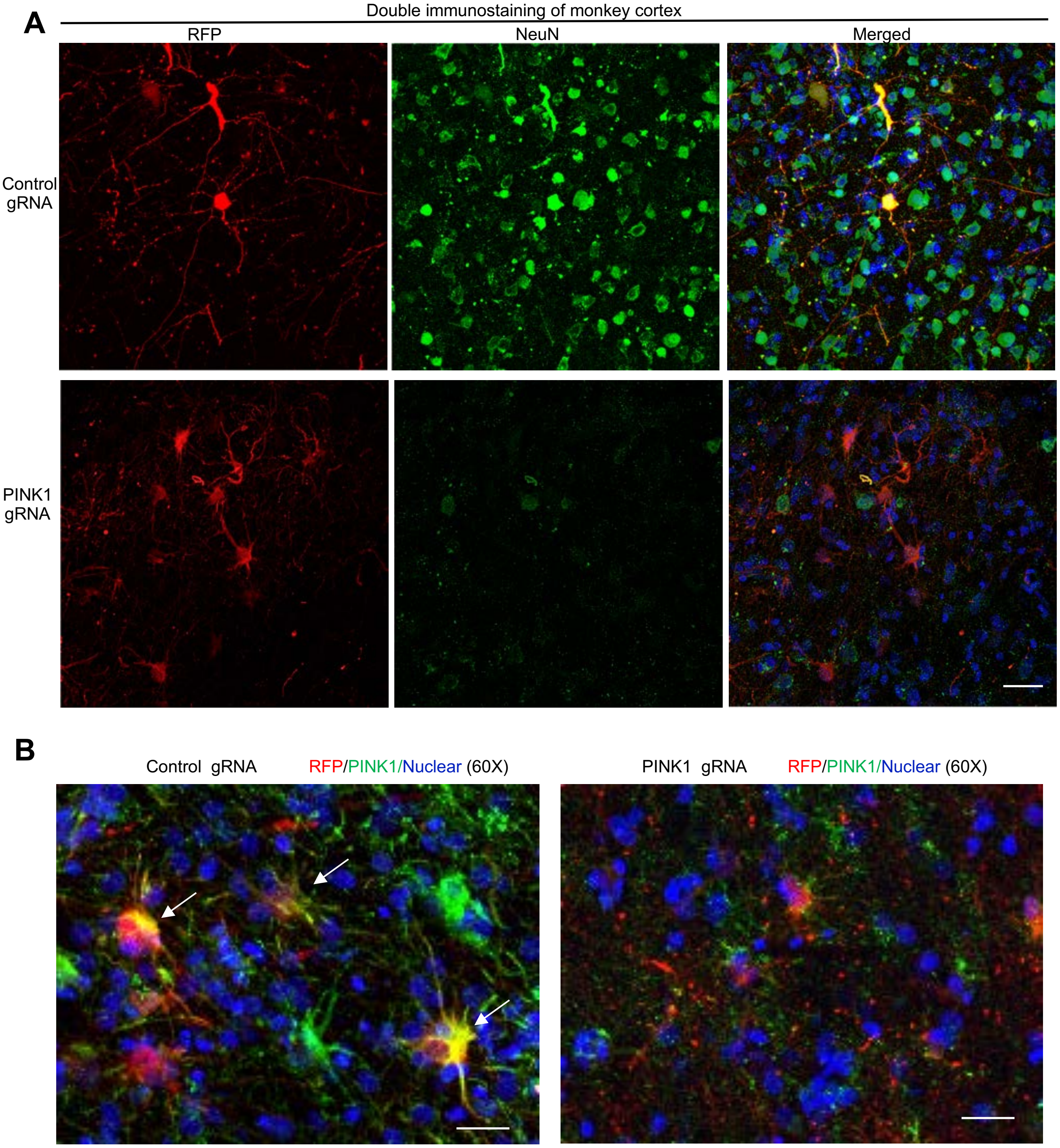

Fig. S5. Targeting *PINK1* in the adult monkey cortex reduced the density of neuronal cells. **(A)** Double immunostaining of the monkey brain cortex injected with AAV-control gRNA-RFP or AAV-PINK1 gRNA-RFP with AAV-Cas9. RFP immunostaining indicates AAV transduced cells and NeuN immunostaining indicates neuronal cells. (**B**) Merged images showing that AAV-control RNA-infected cells (arrows) also express NeuN, which are absent in the merged image from the AAV-PINK1 gRNA-injected brain region. Scale bars: 20 µm.

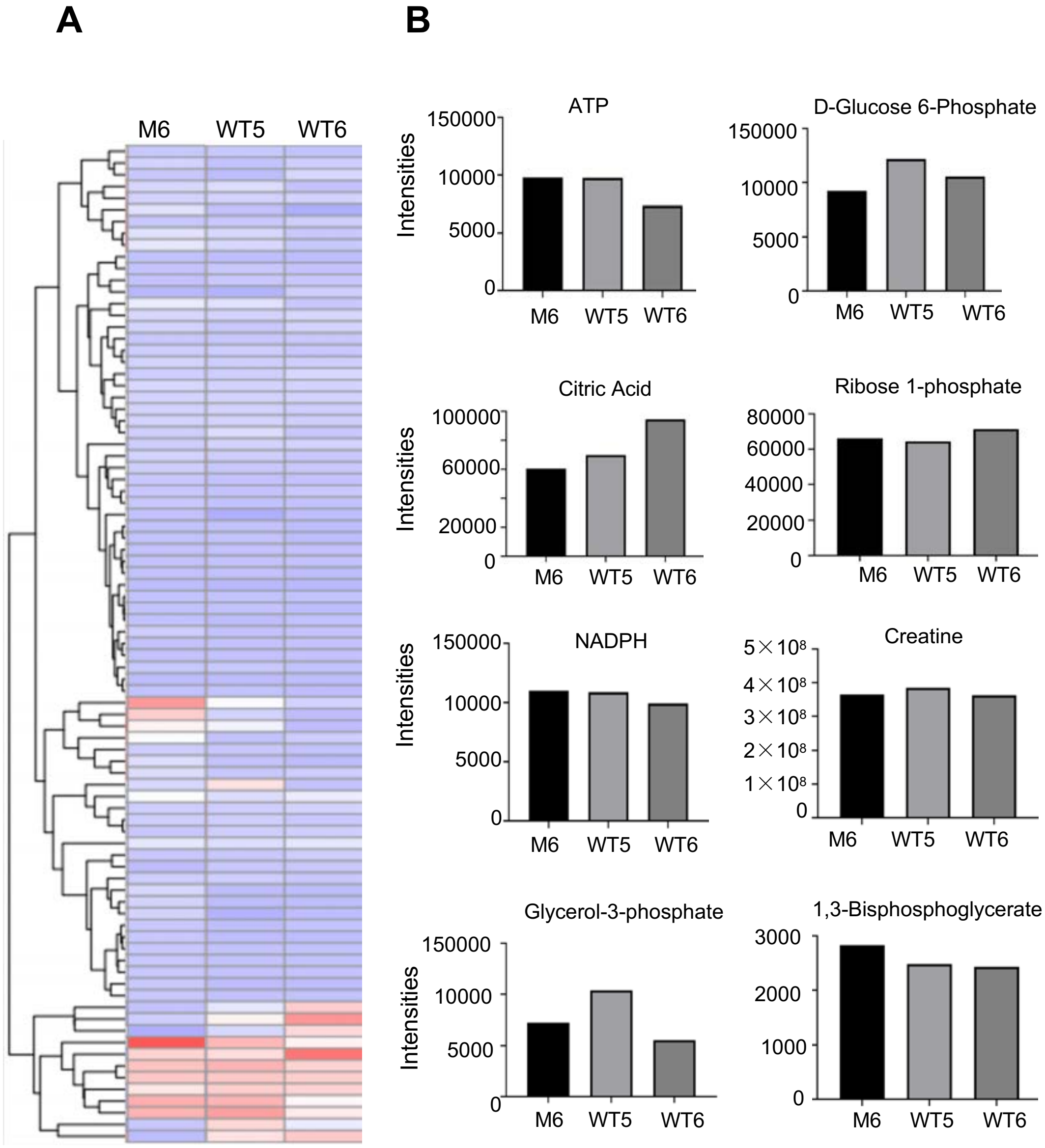

Fig. S6. Metabolomics of monkey cortex tissues. (**A**) Heatmap of metabolomics of M6 and two WT (WT5 and WT6) monkey cortex tissues. (**B**) The relative levels of representative metabolites related to mitochondrial functions.

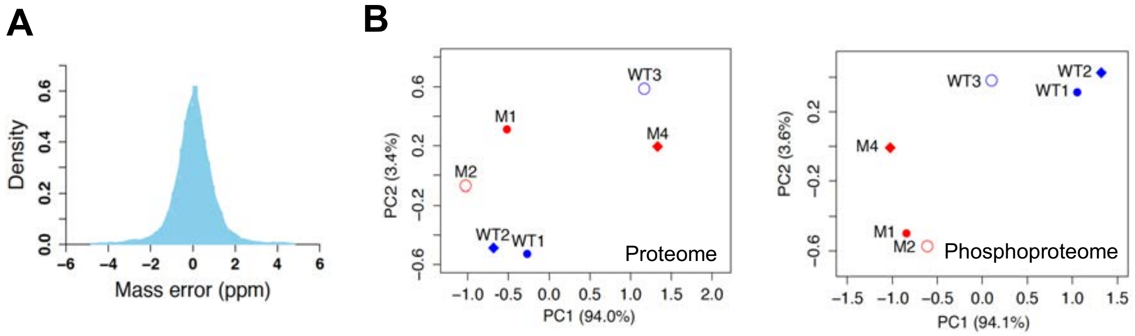

C Changed phosphoproteins and their kinases

Kinase	Substrate	ProteinID	Gene name	Site	Phosphosite sequence	FC (M/WT)	P value
MAPKAPK2	HSP27	P04792	HSPB1	S82	QLsSGVSEIR	0.23	0
CDK2	MARCKS	P29966	MARCKS	T150	AEDGATPSPSNEtPK	0.17	0.01
CDK5	CRMP-1	Q14194	CRMP1	S522	YATPAPSAKsSPSK	0.13	0.03
PKCE	PKCE	Q02156	PRKCE	S368	ALsFDNR	0.24	0.03
GSK3B	PKCE	Q02156	PRKCE	S346	sAPTSPCDQEIK	0.34	0.04
CDK1	SYN3	O14994	SYN3	S470	LSPQGQQPLsPQSG SPQQQR	0.4	0.04
PKCE	PKCE	Q02156	PRKCE	S234	KQETPDQVGsQR	0.3	0.04
P38B	PKCE	Q02156	PRKCE	S350	SAPTsPCDQEIK	0.34	0.05
GSK3B	CRMP-2	Q16555	DPYSL2	T514	TVtPASSAK	0.21	0.01
GSK3A,3B	CRMP-2	Q16555	DPYSL2	S518	TVTPASsAKTSPAK	0.11	0.01
PDHK1,2,3,4,PDK1,PDP1	PDHA1	P08559	PDHA1	S293	YHGHsMSDPGVSYR	0.5	0

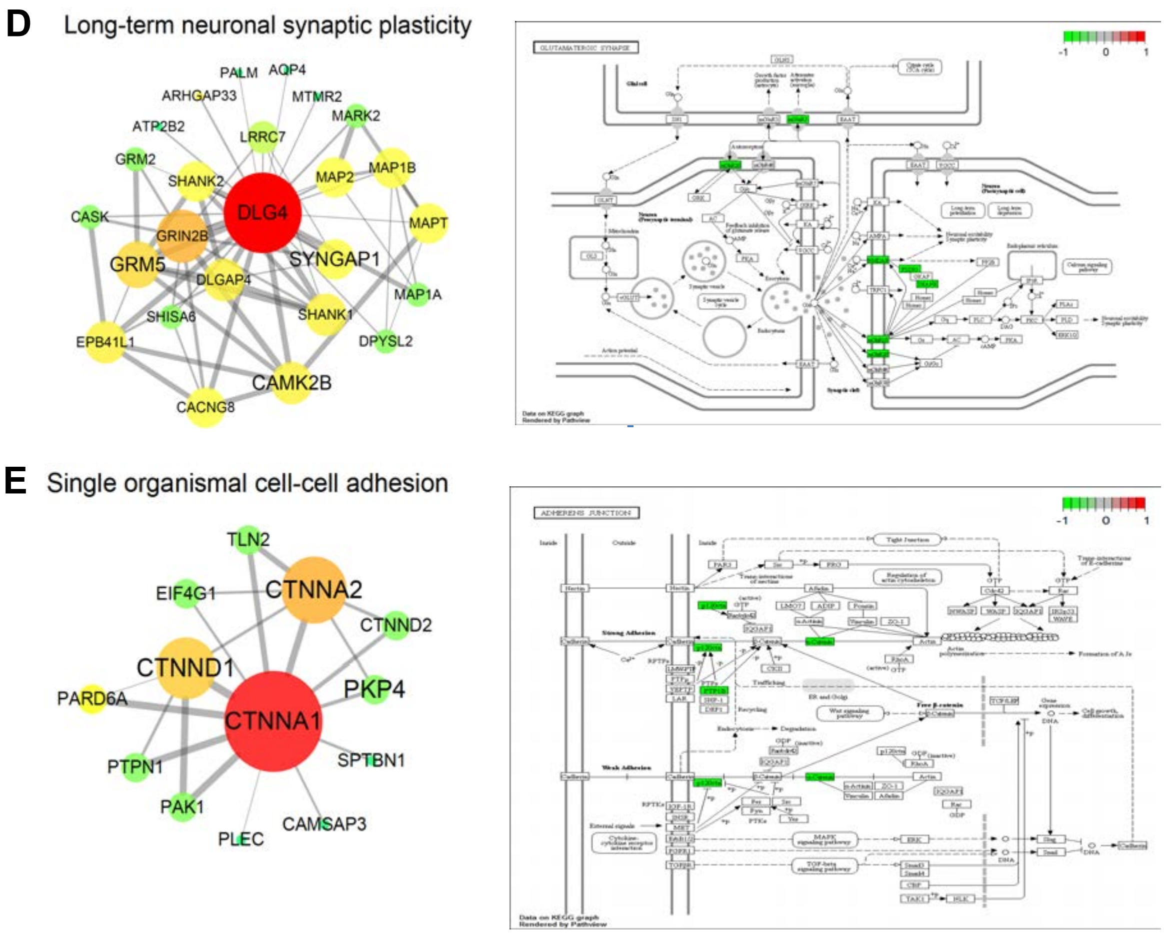

Fig. S7. Additional mass spectrometry data showing the reduced protein phosphorylation in the PINK1 mutant monkey brains. (**A**) Mass accuracy of the phosphopeptide measurement in this study. The X-axis represents mass errors in ppm and the Y-axis represents frequency. (**B**) Principal component analysis of the quantified proteome and phosphoproteome comparing the wild type and mutant monkey cortices. (**C**) Summary of changed phosphosites and their predicted kinases that have been verified in the literature. (**D, E**) Network analysis of proteins with significantly reduced phosphorylation in *PINK1* mutant monkeys shows a network in synaptic plasticity (D) and cell-cell adhesion (E). In the constructed network, the size of the node represents the connectivity of a protein with other proteins, and the thickness of the edge represents the strength of the interaction.

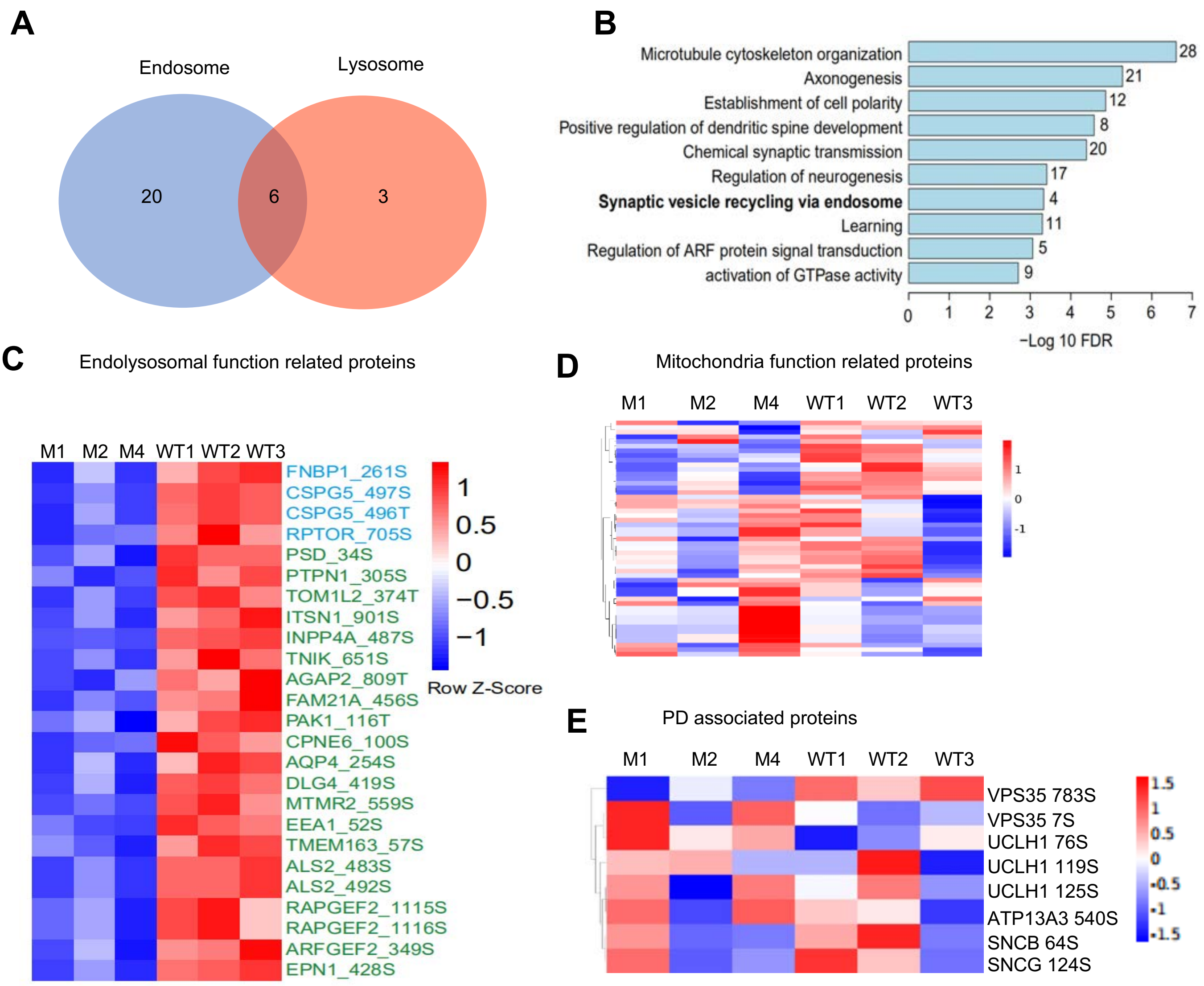

Figure S8. Reduced phosphorylation of endosomal function-related proteins that are also important for neuronal function. (**A**) Venn diagram showing the proteins that are involved in endosome (left circle, 26) and lysosome (right circle, 9), and also the overlap phosphoproteins between endosome and lysosome (middle circle, 6). (**B**) Biological process of changed phosphoproteome showing the enriched proteins of endosome system that are involved in synaptic vesicle function. (**C**) Heatmap of the phosphorylation sites from the endolysosomal proteins, most of which are also important for neuronal function. (**D**) Heatmap of the phosphorylation sites from proteins that are involved in mitochondria function showing most of these phosphorylation sites have no significant changes in *PINK1* mutant monkey cortex. (**E**) Heatmap of the phosphorylation sites from PD associated proteins (VPS35, UCLH1, ATP13A3, SNCB and SNCG) showing no consistent changes in *PINK1* mutant monkey cortex.

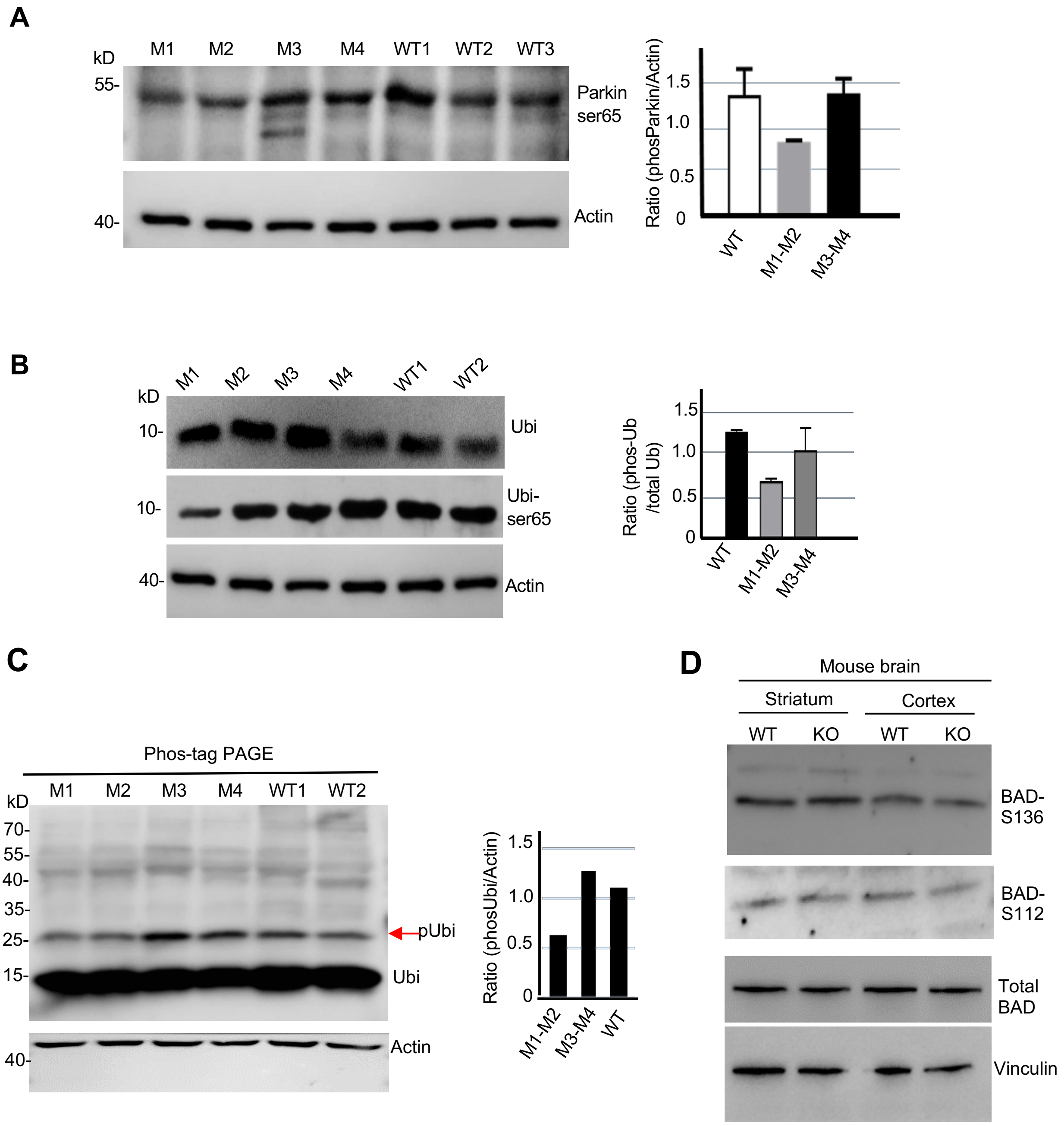

Figure S9. Western blot analysis of protein phosphorylation in the monkey and mouse brains. (**A**) Western blot analysis showing that Parkin-S65 phosphorylation was reduced in M1 and M2 *PINK1* mutant monkey brain cortex tissues but not in M3-M4 brain cortex tissues. The ratios of phosphor-Parkin to actin are presented in the right panel. (**B**) Western blot analysis showing that phosphorylated ubiquitin was reduced in M1 and M2 brain cortical tissues. (**C**) Phos-tag PAGE showing that ubiquitin phosphorylation was reduced in M1 and M2 brain cortical tissues but not in M3 and M4 samples. The ratios of phosphor-ubiquitin to non-phospho-ubiquitin are presented in the right panel. (**D**) Western blotting of mouse brain striatum (Str) and cortex (Ctx) showing that *Pink1* knockout dose not influence BAD phosphorylation at S136 and S112 as compared to wild type (WT) mouse brain tissues.

Supplementary Table 1

Monkey information

Name	Targeted gene	Targeted exons	Sex	Death and age
M1	PINK1	Exon2 and Exon4	M	Postnatal death (166 d)
M2	PINK1	Exon2 and Exon4	F	Postnatal death (188 d)
M3	PINK1	Exon2 and Exon4	M	Postnatal death (166 d)
M4	PINK1	Exon2 and Exon4	M	Postnatal death (166 d)
M5	PINK1	Exon2 and Exon4	M	1.5 yr (sudden death)
M6	PINK1	Exon2 and Exon4	F	3 yr (euthanized)
M7	PINK1	Exon2 and Exon4	M	Gestation day139 (aborted)
WT1	_	_	M	Postnatal death (173 d)
WT2	_	_	F	Postnatal death (158 d)
WT3	_	_	F	Gestation day145 (aborted)
WT4	_	_	M	1 Month (euthanized)
WT5	_	_	M	1.5 yr (euthanized)
WT6	_	_	M	3 yr (euthanized)
WT7	_	_	M	Gestation day135 (aborted)
WT8	_	_	F	Gestation day140 (aborted)
WT9	_	_	M	3 yr (euthanized)
WT10	_	_	M	5 yr (euthanized)
WT11	PINK1	Exon2 and Exon4	M	3 yr (AAV injection then euthanized)
WT12	PINK1	Exon2 and Exon4	M	12 yr (AAV injection then euthanized)
WT13	PINK1	Exon2 and Exon4	M	12 yr (AAV injection then euthanized)
WT14	PINK1	Exon2 and Exon4	M	10 yr (AAV injection then euthanized)

Supplementary Table 2

Information of antibodies and reagents

REAGENT or RESOURCE	SOURCE	IDENTIFIER
**Antibodies**		
Mouse monoclonal Anti-β-actin	Santa Cruz	Cat# 47778
Mouse monoclonal anti-γ-tubulin	Sigma	Cat# T6557
Mouse monoclonal anti-NeuN	Millipore	Cat# MAB377
Rabbit polyclonal anti-GFAP	Millipore	Cat# AB5804
Rabbit polyclonal anti-Doublecortin	Cell signaling	Cat# 4604
Rabbit anti-SNAP25	Cell signaling	Cat# 5308
Monoclonal Anti-CRMP2	Cell signaling	Cat# 9393
Rabbit monoclonal anti-Vinculin	Abcam	Cat# ab129002
Mouse monoclonal anti-Phosphotyrosine	Millipore	Cat# 05-321
Rabbit polyclonal anti-phosphoserine	Millipore	Cat# AB1603
Rabbit polyclonal anti-phosphothreonine	Millipore	Cat# AB1607
Rabbit polyclonal anti-RFP	Rockland	Cat# 600-401-379
Rabbit polyclonal anti-Beclin1	Abcam	Cat# ab55878
Rabbit polyclonal anti-LC3B	Novus biologicals	Cat# NB100-2220
Rabbit polyclonal anti-mTOR	Cell signaling	Cat #2983
Rabbit polyclonal anti-HSP70	Cell signaling	Cat# 4876
Rabbit polyclonal anti-HSP60	Abcam	Cat# ab46798
Rabbit polyclonal anti-Caspase 3	Cell signaling	Cat# 9665
Rabbit polyclonal anti-PINK1	Abcam	Cat# ab23707
Donkey Anti-Rabbit	Jackson Immunolabs	Cat# 715-035-152
Rabbit polyclonal anti-PINK1	Novus biologicals	Cat# BC100-494
Rabbit polyclonal anti-Parkin (phosopho S65)	Abcam	Cat# ab154995
Donkey Anti-Mouse	Jackson Immunolabs	Cat# 715-035-151
Rabbit polyclonal anti-PINK1	Cell signaling	Cat# 6946
Rabbit polyclonal anti-synapsin-1	Cell signaling	Cat# 5297

Rabbit polyclonal anti-Bad	Cell signaling	Cat# 9292
Rabbit polyclonal anti-Bad(Ser112)	Cell signaling	Cat# 5284
Rabbit polyclonal anti-Bad(Ser136)	Cell signaling	Cat# 4366
Rabbit polyclonal anti-Tom20	Santa cruz	SC17764
Rabbit polyclonal anti-VDAC1	Abcam	Ab14734
Rabbit polyclonal anti-Drp1(S616)	Cell signaling	Cat# 3455
Rabbit polyclonal anti-Bcl-XL	Cell signaling	Cat# 2762
sheep polyclonal anti-PINK1	MRC PPU Reagents	S085D (anti-human PINK1, 175-250 aa)
sheep polyclonal anti-PINK1	MRC PPU Reagents	S086D (anti-mouse PINK1, 175-250 aa)
sheep polyclonal anti-PINK1	MRC PPU Reagents	S774C (anti-mouse PINK1, 235-511 aa)
sheep polyclonal anti-PINK1	MRC PPU Reagents	S460C (anti-human PINK1, 125-539 aa)
Rabbit polyclonal anti-CRMP2(T514)	Cell signaling	Cat# 9397
Rabbit polyclonal anti-OPA1	Cell signaling	Cat# 80471
Rabbit polyclonal anti-Mfn1	Cell signaling	Cat# 14739
mouse polyclonal anti-NdufA10	Santa Cruz	SC-376357
Rabbit polyclonal anti-STXBP1(S515)	Abnova	PAB9675
Rabbit polyclonal anti-STXBP1	Abcam	Ab3451
Rabbit polyclonal anti-AKT	Cell signaling	Cat# 4691
Rabbit polyclonal anti-AKT (T308)	Cell signaling	Cat# 13038
**Bacterial and Virus Strains**		
Xl-blue	Stratagene	Cat#200249
**Chemicals, Peptides, and Recombinant Proteins**		
DAPI	Sigma	Cat# D9542
Collagenase IV	Sigma	Cat# C5138
PBS	Hyclone	SH30256
Trypsin-EDTA solution	Sigma	Cat# T4049
DMEM	Hyclone	SH30243.01
FBS	Hyclone	Cat# SH30084.03

Penicillin	Sigma	Cat# P3032
Streptomycin sulfate	Sigma	Cat# S6501
Dimethyl sulfoxide	Sigma	Cat# D8779
CCCP	Sigma	Cat# c2759
MG132	Sigma	CatM7449
**Critical Commercial Assays**		
VECTASTAIN Elite ABC Kits	Vector	Cat# PK-2200
mMESSAGE mMACHINE T7 kit	Ambion	Cat# AM1344
MAXIscript T7	Ambion	Cat# AM1312
ECL^{TM} prime western blotting detection Kit	Fisher scientific	Cat# 45-002-401
**Deposited Data**		
Monkey gene definitions for PINK1	NCBI	https://www.ncbi.nlm.nih.gov/gene/706037
Human gene definitions for PINK1	NCBI	https://www.ncbi.nlm.nih.gov/gene/65018
Mouse gene definitions for PINK1	NCBI	https://www.ncbi.nlm.nih.gov/gene/68943
**Experimental Models: Cell Lines**		
HEK 293 cell	Li lab at Jinan University	N/A
N2A cell	Li lab at Jinan University	N/A
SHY5Y cell	Li lab at Jinan University	N/A
Human fibroblast cells	Xiangya Hospital	N/A
**Experimental Models: Organisms/Strains**		
PINK1 mutant monkeys	This paper	N/A
PINK1 KO mice	This paper	N/A
Primary cultured monkey cortical cells	This paper	N/A

**Oligonucleotides**		
Monkey PINK1-Exon2 sgRNA	Thermo Fisher, Guangzhou,China	GGCTGGAGGAGTATCTGATAGGG
Monkey PINK1-Exon4 sgRNA	Thermo Fisher, Guangzhou,China	CCGGGTTCTCCGCGCTTTCACC
Monkey Htt-T1 sgRNA	Thermo Fisher, Guangzhou,China	CCGGGTTCTCCGCGCTTTCACC
Monkey Htt-T3 sgRNA	Thermo Fisher, Guangzhou,China	GGCCTTCATCAGCTTTTCCAGGG
T7-sgRNA Sense Primer	Thermo Fisher, Guangzhou,China	GAAATTAATACGACTCACTATA
T7-sgRNA Anti-sense Primer	Thermo Fisher, Guangzhou,China	AAAAAAAGCACCGACTCGGTGCCAC
Monkey PINK1-Exon2 primer-F	Thermo Fisher, Guangzhou,China	CCAGGCTGAGCAGTAGAA
Monkey PINK1-Exon2 primer-R	Thermo Fisher, Guangzhou,China	TGAACCTAATCCCTGGGTGA
Monkey PINK1-Exon4 primer-F	Thermo Fisher, Guangzhou,China	CCAGGCTGAGCAGTAGAA
Monkey PINK1-Exon4 primer-R	Thermo Fisher, Guangzhou,China	TGAACCTAATCCCTGGGTGA
Monkey PINK1-Exon3 primer-F	Thermo Fisher, Guangzhou,China	ACACAATGAGCCAGGAGCTG
Monkey PINK1-Exon3 primer-R	Thermo Fisher, Guangzhou,China	CAGAGGGCACTGACCTGTAA
Mouse and Monkey PINK1 Exon1-2 primer-F	Thermo Fisher, Guangzhou,China	TCGGCCTGTCAGGAGATCCAGGCAAT
Mouse and Monkey PINK1 Exon1-2 primer-R	Thermo Fisher, Guangzhou,China	GGCATGGTGGCTTCATACACAGCGGCA
Mouse and Monkey PINK1 Exon 6-7 primer-F	Thermo Fisher, Guangzhou,China	CCATCGCCTATGAAATCTTTGGGCT
Mouse and Monkey PINK1 Exon 6-7 primer-R	Thermo Fisher, Guangzhou,China	CTCTTGCTGGCCTCTCGCTGGAGC
Mouse and Monkey β-actin primer-F	Thermo Fisher, Guangzhou,China	GAAGATCAAGATCATTGCTCCTC
Mouse and Monkey β-actin primer-R	Thermo Fisher, Guangzhou,China	CTGCTTGCTGATCCACATCTGCTG

Mouse PINK1-Exon2 sgRNA	Thermo Fisher, Guangzhou,China	GTCTGGAGGATTATCTGATAGGG
Mouse PINK1-Exon4 sgRNA	Thermo Fisher, Guangzhou,China	CCGGGTTTTCCGCGCCTTCACCT
Mouse PINK1 Exon2 genotyped primer-F	Thermo Fisher, Guangzhou,China	CTCCCCACTCTTGTGTTTGCTATGT
Mouse PINK1 Exon2 genotyped primer-R	Thermo Fisher, Guangzhou,China	CAGTTGCTGCTCAGAGTAGTTCACA
Mouse PINK1 Exon4 genotyped primer-F	Thermo Fisher, Guangzhou,China	CACCATGTGAGATGGATAGATGGGC
Mouse PINK1 Exon4 genotyped primer-R	Thermo Fisher, Guangzhou,China	AAGTTAGCTGGCACTGAAAGAGGAC
**Software and Algorithms**		
GraphPad Prism 8	Graphpad Software	www.graphpad.com
SnapGene 3.0	GSL Biotech LLC	www.snapgene.com
Stereo Investigator 5.4.3	Micro Bright Field Bioscience	www.mbfbioscience.com
Image J	National Institutes of Health	https://imagej.nih.gov/ij/docs
**Other**		
Axio Imager A2	Zeiss	Carl Zeiss, Germany
Axio Imager 2	Zeiss	Carl Zeiss, Germany
Zeiss LSM 800 Confocal Laser Scanning Microscope	Zeiss	Carl Zeiss, Germany
MRI scanner	Siemens	Erlangen, Germany
FV3000 Microscope	Olympus	Japan
Spin SR confocal system	Olympus	Japan
